# Unveiling the pathways of Xuanbai Chengqi Decoction in obese asthma: from immune modulation to microbial restoration

**DOI:** 10.3389/fnut.2026.1710739

**Published:** 2026-01-23

**Authors:** Ke Lu, Chen Li, Qingxiang Zhang, HongXiang Li, Chen Ding, Lu Zhang, Feng Cao

**Affiliations:** 1 School of Basic Chinese Medicine (Qihuang College), Guizhou University of Traditional Chinese Medicine, Guiyang, Guizhou, China; 2 School of Health Care, Guizhou University of Traditional Chinese Medicine, Guiyang, Guizhou, China; 3 School of Traditional Chinese Medicine, Shandong University of Traditional Chinese Medicine, Jinan, Shandong, China; 4 Key Laboratory of Traditional Chinese Medicine Classical Theory, Ministry of Education, Shandong University of Traditional Chinese Medicine, Jinan, Shandong, China; 5 School of Health, Shandong University of Traditional Chinese Medicine, Jinan, Shandong, China

**Keywords:** lung and intestine, mechanism, obese asthma, Th17/Treg, Xuanbai Chengqi Decoction (XBQCD)

## Abstract

**Background:**

Obesity asthma is a unique asthma phenotype, which has the characteristics of aggravation of clinical symptoms, change of immune response, and resistance to standard treatment. Obese asthma, as a clinical refractory asthma type, urgently needs effective and side-effect-free treatment. Xuanbai Chengqi Decoction (XBCQD) is a traditional Chinese medicine prescription widely used in the treatment of lung diseases, including asthma in China. However, the efficacy and mechanism of obese asthma remain to be explored.

**Objectives:**

To elucidate the therapeutic effect of XBCQD on obese asthma and reveal its mechanism.

**Methods:**

Network pharmacology was used to predict the potential therapeutic targets and pathways of XBCQD in the treatment of obese asthma. We established a mouse model of obese asthma by feeding a high-fat diet combined with intraperitoneal injection of ovalbumin (OVA) to induce sensitization, and then intervened with intragastric administration of high, medium, and low doses of XBCQD. During the modeling period, lung function and body weight of mice were used to evaluate the preparation of the obese asthma model. H&E staining, RT-qPCR, ELISA, Western blot, and flow cytometry were used to quantify Th cell subsets, 16S rRNA sequencing was used to determine microbial composition, and GC/MS was used to detect the content of short-chain fatty acids in intestinal contents to explore the mechanism of Xuanbai Chengqi Decoction on obese asthma.

**Results:**

Network pharmacology showed that XBCQD may improve obese asthma by affecting core targets such as IL-6, TNF, and Caspase1, and through signaling pathways such as the IL-17 signaling pathway, AGE-RAGE signaling pathway, TNF signaling pathway, and Th17 cell differentiation. Experimental studies have found that XBCQD can alleviate the symptoms of obese asthma and lung inflammation, reduce serum IgE, reduce the expression of IL-6, IL-17, and IL-23 in serum, to reduce lung inflammation induced by obese asthma in mice; flow cytometry of spleen tissue showed that XBCQD reduced the proportion of Th17 cells and restored the proportion of Treg cells. Proteomics showed that XBCQD inhibited the expression of NLRP3, Caspase-1, and IL-1β by up-regulating the expression of GPR43, thereby inhibiting Th17-related protein RORγt and restoring Treg-related protein Foxp3, thereby regulating immune imbalance. At the same time, XBCQD restored the intestinal microbial species, and restored the beneficial bacteria such as *Dubosiella*, *Akkermansia_muciniphila*, *Rikenella*, which were reduced in obese asthmatic mice, and increased the content of acetic acid, propionic acid, and butyric acid in intestinal flora metabolites.

**Conclusion:**

XBCQD regulates Th17/Treg immune imbalance in obese asthma by improving intestinal microecology and regulating SCFAs/GPR43/NLRP3 pathway. These findings provide new pharmacological evidence for its clinical application in obese asthma.

## Graphical abstract

**Figure:**
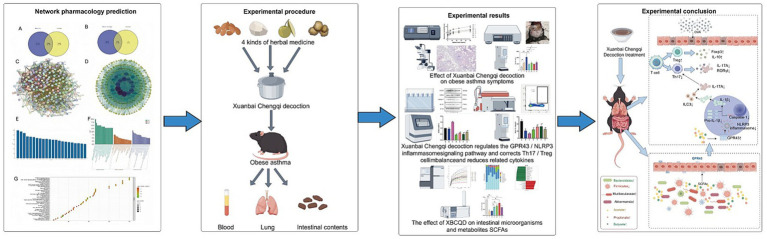

## Introduction

In recent decades, the global prevalence of obesity and allergic diseases has been on the rise, with obese asthma (OA) being recognized as a distinct phenotype of asthma according to the 2014 Global Initiative for Asthma (GINA) guidelines (1). Research indicates that obesity can influence the pathophysiological processes of allergic respiratory diseases through mechanisms such as systemic inflammatory infiltration, alterations in immune response, and adjustments in respiratory mechanics (2–4). Compared to individuals of normal weight, those with obesity-related chronic inflammation may experience exacerbated airway inflammation, worsened symptoms, and an increased risk of treatment resistance in allergic conditions. Currently, asthma treatment primarily involves the use of glucocorticoids and anticholinergic drugs (5). However, long-term use of these medications can lead to side effects and disease recurrence, with limited patient adherence (6, 7). Consequently, there is an urgent need to further investigate the complex mechanisms underlying obesity and allergic airway inflammation and to develop safe and effective treatment strategies to alleviate the clinical burden of allergic airway diseases in obese individuals.

Atopic asthma represents the predominant form of T2 high-phenotype asthma, with its pathogenesis intricately linked to a Th2-type immune response resulting from a compromised epithelial barrier (8, 9). Obesity exacerbates T2 inflammation and facilitates the shift of the asthma immunophenotype towards a Th17-dominated T3 type. This shift is mediated through the induction of cytokines such as IL-23 and IL-17, which disrupt the Th17/Treg immune equilibrium, promote neutrophil infiltration, contribute to airway remodeling, and enhance disease resistance (10–12). Within this context, the activation of the NLRP3 inflammasome and the secretion of leptin by adipose tissue serve as critical mediators (13). The NLRP3 inflammasome intensifies inflammation by modulating IL-1β release, while leptin fosters Th17 cell differentiation and activates associated inflammatory pathways (14–17). The intestinal tract is the organ with the highest colonization density of human microbiota, and its microbial composition and dysfunction have been demonstrated to regulate pulmonary immune homeostasis via the “gut-lung axis” (18, 19). Obesity is frequently associated with a reduction in intestinal microbiota diversity and an increased ratio of *Firmicutes* to *Bacteroides* (20). The metabolites produced by these microbiota, such as short-chain fatty acids (SCFAs), can influence distal lung immunity through systemic circulation (21). SCFAs have the potential to promote regulatory T cell (Treg) generation by modulating Foxp3 acetylation and inhibiting dendritic cell activation, thereby regulating the Th17/Treg balance (22, 23). A decrease in the abundance of SCFA-producing bacteria is linked to an elevated risk of asthma (24, 25). Given the pivotal regulatory role of the gut microbiota-immune interaction mediated by the gut-lung axis in the Th17/Treg imbalance observed in obesity-related asthma, a comprehensive analysis of the molecular mechanisms underlying this pathway could provide a significant theoretical foundation for developing novel therapeutic strategies targeting gut microbiota or immune balance in obesity-related asthma.

For millennia, traditional Chinese medicine (TCM) has been employed by the Chinese population for the prevention and treatment of various complex diseases, adhering to the principle of comprehensive and holistic regulation of human yin and yang (26). This approach has demonstrated significant efficacy and continues to be extensively utilized in contemporary practice. Research indicates that TCM possesses distinct advantages in managing complex diseases, particularly in the treatment of obese asthma, due to its multi-component, multi-target, and multi-channel effects on pathological conditions, coupled with its low toxicity and minimal side effects (27). Xuanbai Chengqi Decoction is a traditional herbal formulation frequently employed in the treatment of pulmonary disorders. The formulation comprises Shi Gao(*Gypsum Fibrosum*), Da Huang(*Rheum officinale* Baill), Ku Xing Ren (*Prunus armeniaca* L), and Gua Lou (*Trichosanthes kirilowii* Maxim); the specific formula is shown in Table 1. It operates through the principles of releasing pulmonary qi and promoting intestinal peristalsis to address respiratory conditions such as asthma. We hypothesize that Xuanbai Chengqi Decoction may facilitate the holistic regulation of pulmonary and intestinal systems by modulating the inflammatory response in the lungs and enhancing the intestinal microbiota. Consequently, this study utilizes Xuanbai Chengqi Decoction and obese asthmatic murine models to investigate its therapeutic efficacy, underlying mechanisms, and potential pathways. This is achieved by integrating network pharmacology predictions with *in vivo* experimental methodologies. The objective of this research is to contribute novel insights into the prevention and management of obesity-related asthma and to establish a foundation for the effective application of Xuanbai Chengqi Decoction as an intervention strategy.

**Table 1:** The composition of XBCQD.

Latin name	Chinese name	Dosage	Source
*Gypsum Fibrosuum*	Shi Gao	15 g	Shandong Xinzhonglu Traditional Chinese Medicine Hospital Co., Ltd.
*Rheum officinale* Baill	Da Huang	9 g	Shandong Xinzhonglu Traditional Chinese Medicine Hospital Co., Ltd.
*Prunus armeniaca* L	Ku Xing Ren	6 g	Shandong Xinzhonglu Traditional Chinese Medicine Hospital Co., Ltd.
*Trichosanthes kirilowii* Maxim	Gua Lou	4.5 g	Shandong Xinzhonglu Traditional Chinese Medicine Hospital Co., Ltd.

## Materials and methods

### Network pharmacology analysis

In this study, the active components of the traditional Chinese medicine systems pharmacology database and analysis platform (TCMSP),^1^ input keywords: Shi Gao (*Gypsum Fibrosum*), Da Huang (*Rheum officinale* Baill), Ku Xing Ren (*Prunus armeniaca* L), and Gua Lou (*Trichosanthes kirilowii* Maxim) were used for searching. *Gypsum Fibrosuum* belongs to the sulfate minerals and is not included in the TCMSP database. Therefore, it is not included in the screening range. The oral bioavailability (OB) ≥ 30% and drug similarity (DL) ≥ 0.18 were used as conditions for screening, and the components of related pharmacological effects were supplemented as potential active components in combination with the literature. The SDF structure of the above components was obtained from the Pubchem database^2^ and saved in Canonical SMILES format. The SMILES format file obtained above was imported into the Swiss Target Prediction database.^3^ “Human” was used as the research species, and the score of intestinal absorption (GI) was “high.” The drug-likeness was screened by at least two “Yes,” so as to obtain the potential targets of the chemical components of Xuanbai Chengqi Decoction. The target of the active components of Xuanbai Chengqi Decoction was obtained by screening with Probability>0 as the condition. Human gene annotation and normalization of protein targets were performed using the Uniprot database.^4^ Using “asthma” and “obesity” as keywords, GeneCards,^5^ online Mendelian Inheritance in Man (OMIM),^6^ Disgenet database,^7^ and Drugbank database^8^ were used to search for their disease targets. The results were imported into Venny2.1 online software mapping tool platform,^9^ to determine the intersection target of obesity-asthma disease. The obtained potential target genes of Xuanbai Chengqi Decoction-obesity related asthma were input into the STRING database.^10^ To determine the intersection target of obesity-asthma disease, the potential target genes of Xuanbai Chengqi Decoction-obesity-related asthma were input into the STRING database, and the organism was selected as “*Homo sapiens*” to obtain the PPI network to present the direct and indirect regulatory relationship between potential target genes. Each node in the PPI network represents a protein, and the edge represents a protein–protein association, that is, the association of common functions between potential target genes. These results were imported into Cytoscape 3.8.0 to construct and analyze a visual PPI network containing topological parameters for the target protein. The key targets were imported into Metascape for gene ontology (GO) function and Kyoto encyclopedia of genes and genomes (KEGG) pathway enrichment analysis. Significantly enriched and obesity-related pathways and biological processes were obtained. The results were plotted with the micro-bioinformatics online drawing tool platform.^11^

### Experimental animals

C57BL/6 J female mice, weighing 14-16 g (4 weeks old), obtained from Beijing Vital River Laboratory Animal Technology Co., Ltd. [SCXK (Beijing) 2021-0006]. The mice were fed in the Animal Experimental Center of Shandong University of Traditional Chinese Medicine (SPF grade) [SYXK (Shandong) 2022-0009] under controlled conditions (12-h light–dark cycle, 22–24 °C) and free access to food and water. All experiments were approved by the Animal Protection Committee of Shandong University of Traditional Chinese Medicine in April 2023 (experimental animal ethics batch number: SDUTCM20230406003).

### Experimental animal grouping and obesity model construction

Before the initiation of the experimental procedures, all mice underwent a one-week adaptive feeding period to acclimate to the experimental conditions. Subsequently, 70 mice were randomly assigned to two groups: the normal control group (*n* = 10) and the model group (*n* = 60). The normal control group received a standard diet suitable for SPF mice, while the model group was administered a high-fat diet for a duration of 10 weeks. The body weight of the mice was monitored and recorded weekly. The establishment of the obesity model was confirmed when the body weight of the mice in the model group exceeded 20% of the average body weight of the normal control group. Following this confirmation, the high-fat diet was continued until the conclusion of the 10 weeks (day 70).

Based on the established success criteria for the obesity model, the mice in the model cohort were subsequently randomized into six distinct groups, with each group comprising ten mice (*n* = 10): the obese model group (f group), the obese asthma model group (fa group), the low-dose Xuanbai Chengqi Decoction group (xl group), the medium-dose Xuanbai Chengqi Decoction group (xm group), the high-dose Xuanbai Chengqi Decoction group (xh group), and the montelukast sodium group (ml group).

### Asthma model sensitization and excitation

On days 71 and 78, mice in the fa, xl, xm, xh, and ml groups underwent sensitization via intraperitoneal injection, receiving 0.2 mL of an ovalbumen (OVA) sensitizing solution, which contained 20 μg of OVA and 2 mg of aluminum hydroxide. In contrast, mice in the n and f groups were administered an equivalent volume of normal saline as a control. From days 85 to 105, spanning a period of 21 consecutive days, the sensitized groups were subjected to aerosolized challenges using 1% OVA solution for inhalation, lasting 20–30 min daily. During this period, mice in the n and f groups were exposed to the same conditions with normal saline.

### Drug preparation and administration plan

Xuanbai Chengqi Decoction (XBCQD) granules were procured from Shandong Xinzhonglu Hospital. The dosage was adjusted for mice based on the equivalent dose for a 60 kg adult. Three concentration gradients were prepared by dilution with distilled water: a high dose of 1.04 g/mL, a medium dose of 0.52 g/mL, and a low dose of 0.26 g/mL. Montelukast sodium, with a specification of 10 mg per tablet (trade name Shunningan, H20183239), was crushed and dissolved in 20 mL of normal saline to achieve a concentration of 0.0005 g/mL.

**Figure 1:**
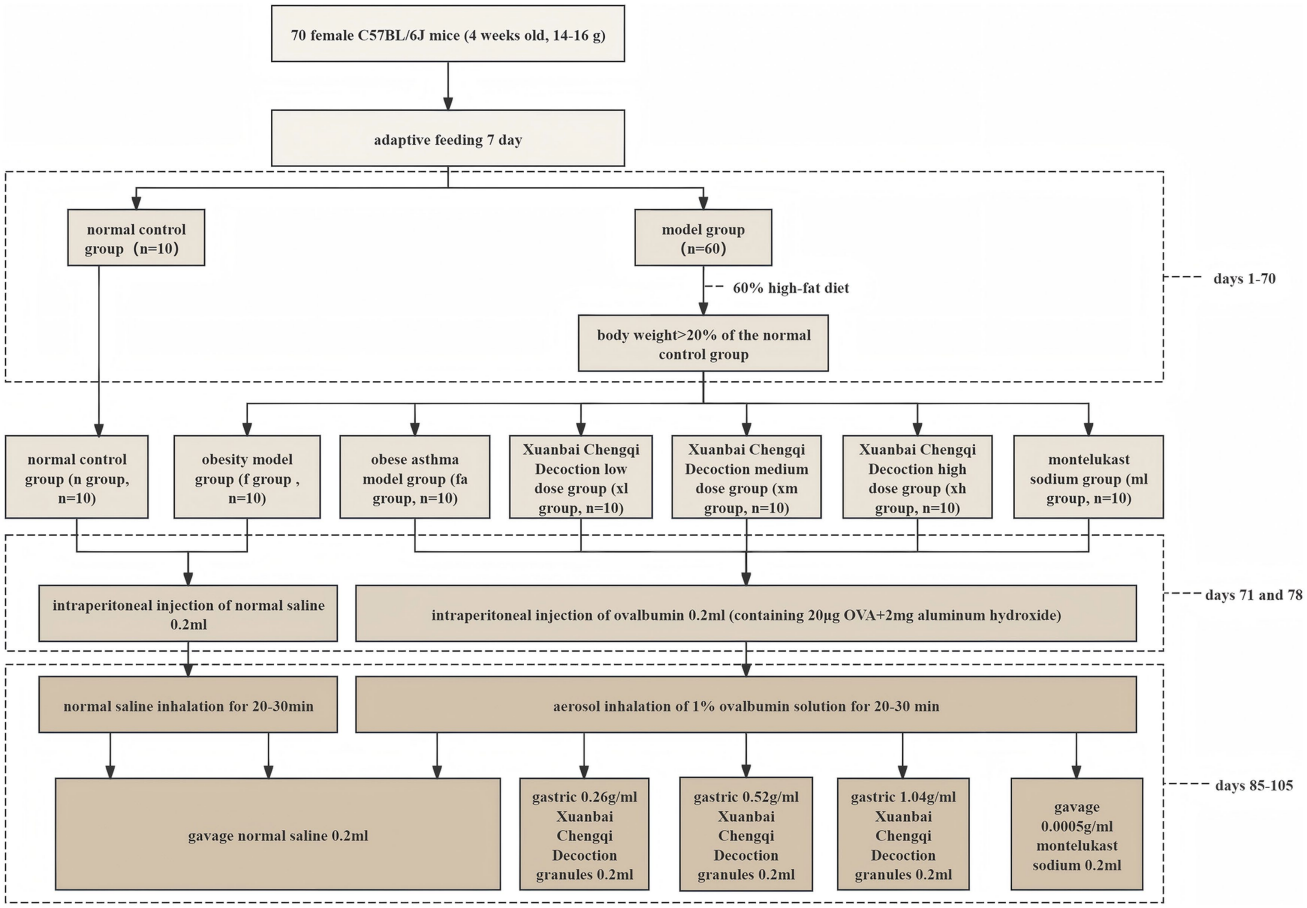
The detailed method of model construction.

**Figure 2:**
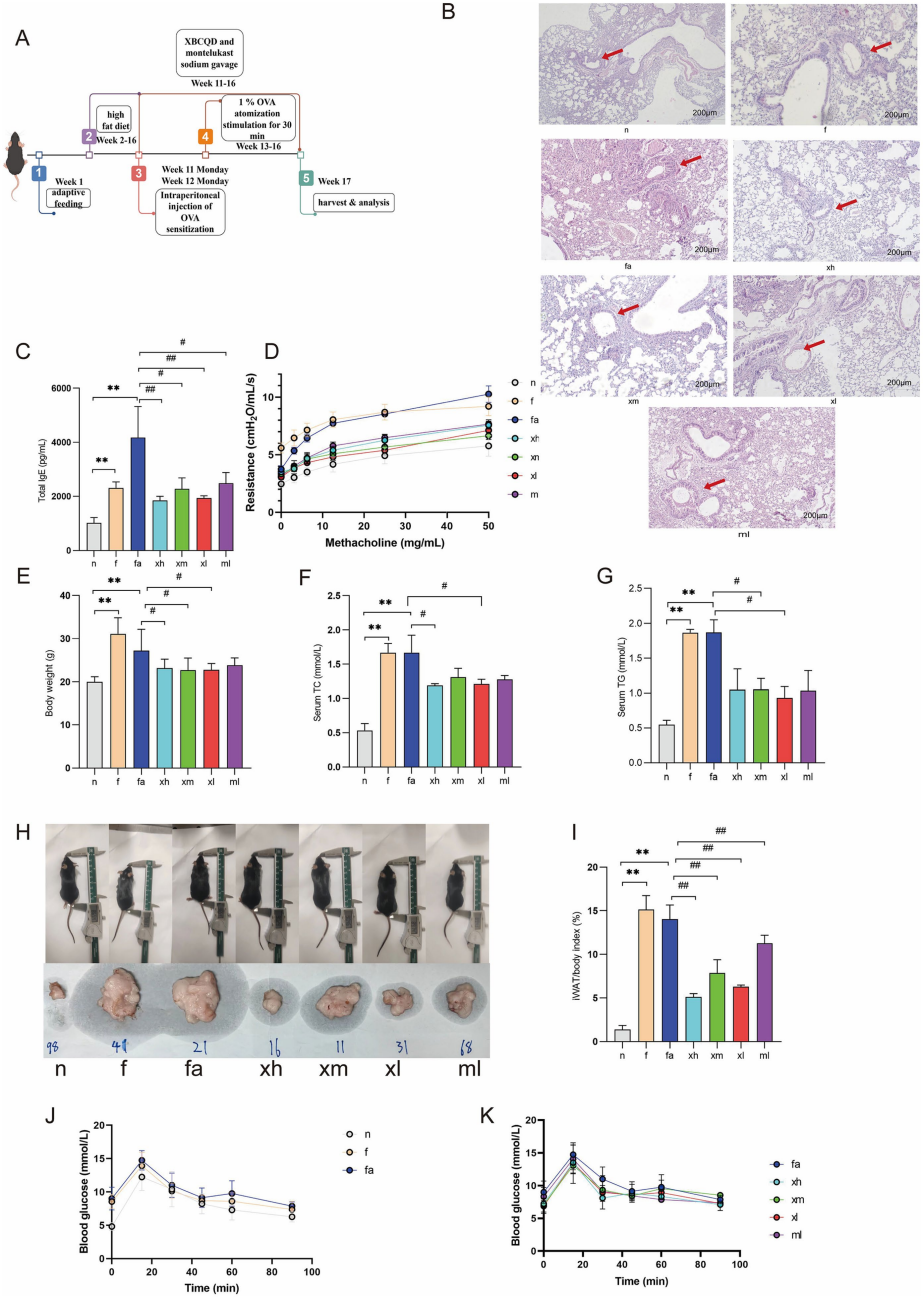
XBCQD treatment improved obese asthma in mice. **(A)** The timeline of the animal test. **(B)** Standard images of HE-stained lung tissues (200×) and inflammation scores, with arrows pointing to areas of inflammatory infiltration. **(C)** Changes of IgE level in BALF of mice (*n* = 5). **(D)** Changes of sRAW level in mice (*n* = 5). **(E)** Weight changes in mice (*n* = 10). **(F,G)** Changes of blood lipid (TC, TG) levels (*n* = 5). **(H,I)** Fat index level changes (*n* = 5). **(J,K)** ACU of OGTT (*n* = 5) (*p < 0.05, **p < 0.01; as compared to the *n* group. #*p* < 0.05, ##*p* < 0.01; as compared to the fa group).

From days 85–105, coinciding with 21 days of asthma induction, mice in each experimental group received daily oral gavage of 0.2 mL. Specifically, the n, f, and fa groups were administered an equivalent volume of normal saline. In contrast, the XH group received an intragastric dose of Xuanbai Chengqi Decoction at a concentration of 1.04 g/mL, the xm group at 0.52 g/mL, and the XL group at 0.26 g/mL. The ml group was administered montelukast sodium at a concentration of 0.0005 g/mL via intragastric gavage. Before administration, all solutions were heated to 36 °C using a constant temperature water bath to prevent thermal shock from affecting the physiological state of the mice. The detailed methodology for model construction is illustrated in Figure 1 and Figure 2A.

### Evaluation of airway resistance in obese asthma

A small animal non-invasive airway resistance detection system (Finepointe-NAM; BUXCO) was used to evaluate the lung function index of mice, and the special airway resistance (sRAW) index was mainly collected. After calibrating the tightness of the cavity, the test was started. The mice were placed in the cavity and fixed. The mouse’s nose was fixed with a rubber membrane to form a relatively closed space, and ventilation was opened. Acetylcholine (Mch, dark) solution 0 mg/mL (PBS), 6.25 mg/mL, 12.5 mg/mL, 25 mg/mL, and 50 mg/mL were added in accordance with the instructions. The respiratory rate and signs of the mice were closely observed, and the results were recorded.

### Pathological evaluation of lung tissue in obese asthma

On day 106, all mice were sacrificed by cervical dislocation. First, we ligated the left lower lung and removed it after complete bronchoalveolar lavage. The right lung tissue was taken out, and the specimens were fixed in formalin buffer, embedded in paraffin, cut into 3 μm sections, and stained with hematoxylin and eosin (HE). To assess airway inflammation and mucus production in lung tissue. The pathological changes were observed under a 200-fold optical microscope.

### Fat index

The adipose tissue on the left and right sides of the abdomen of the mice was separated, and the adipose tissue was taken out and rinsed with normal saline. After the filter paper had dried, the weight was measured and used to calculate the fat index. Fat index formula: fat index (%) = fat weight*100/weight.

### Serum TC, TG detection

0.5–1.0 mL of blood was collected from the posterior orbital venous plexus. The blood was naturally coagulated for 10–20 min at room temperature, and centrifuged at 3000 ×*g* for 20 min. The supernatant was collected and stored at −80 °C for later use. Total cholesterol assay kit (A111-1-1, Nanjing Jiancheng Bioengineering Institute) and Triglyceride assay kit (A110-1-1, Nanjing Jiancheng Bioengineering Institute) were used to detect serum TC and TG levels. The steps were as follows: blank holes, calibration holes, and sample holes were set in 96-well plates. Add 2.5 μL distilled water and 250 μL working solution to the blank well; 2.5 μL calibrator and 250 μL working fluid were added into the calibration hole. 2.5 μL sample and 250 μL working solution were added to the sample hole, mixed, and incubated at 37 °C for 10 min, the wavelength was 500 nm, and the absorbance of each hole was measured by a microplate reader.

### Enzyme-linked immunosorbent assay

In order to measure the level of cytokines in the lung tissue, the frozen alveolar lavage fluid was stored in a refrigerator at −80 °C to obtain the supernatant for analysis. The levels of IgE, IL-17A, and IL-10 in the alveolar lavage fluid of mice were detected by ELISA kit (JL12885, JL20251, JL20242, Shanghai, China). The detection method follows the manufacturer’s instructions. The extinction was measured at 450 nm on a microplate reader ELISA microplate reader.

### Flow cytometry

The fresh spleen tissue was crushed, centrifuged, and made into cell suspension. According to the ratio of 1:1,000, 1 μL of activated cell antibody PMA, Ionomycin, and protein transport inhibitors brefeldin A and Monensin were added to the sample tube. Incubation in the incubator at 37 °C for 5–6 h in the dark, and centrifuging at 4 °C for 1,500 rpm/5 min. The surface antibody CD4 and CD25 diluents were prepared, and the diluents were prepared using 4 °C pre-cooled PBS and FBS. The preparation volume was prepared according to (sample number × 100 μL PBS containing 2% FBS). Add surface antibody CD4, CD25 to the diluent, the ratio of surface antibody and diluent was 1:100 and 1:50, respectively. The surface antibody CD4, CD25, and diluent were fully mixed using a pipette to ensure that the surface antibody CD4, CD25, and diluent were mixed evenly. They were added to the corresponding sample centrifuge tube, and 100 μL of surface antibody CD4 and CD25 diluent was added to each sample centrifuge tube. After completion, incubation at 4 °C for 45 min, centrifuge to remove the upper surface antibody CD4 diluent. According to the volume of 1 mL membrane-breaking buffer for each sample, 10 × Permeabization Buffer buffer (1:9 deionized water dilution) was diluted with deionized water to prepare 1 × Permeabization Buffer buffer. Remove the upper buffer. According to each sample tube, 100 μL of membrane-breaking fluid was added and mixed evenly, so that the cells could complete the membrane-breaking. (Fixation/permeabilization and ebioscience Fixation/Perm Diluent ratio is 1:3). The cells were incubated at 4 °C in the dark to complete the membrane rupture. The sample tube was added with 1 mL membrane-breaking buffer (1 × Permeabization Buffer), and the cells in the sample tube were uniformly mixed to remove the upper buffer. Add 1 mL membrane-breaking solution to make the cell precipitate mixed evenly, centrifuged at 1,500 rpm/5 min, and remove the upper membrane-breaking solution. Each sample tube was added with 500 μL PBS and mixed evenly for detection.

### Western blotting

Lung tissue was dissolved in RIPA buffer containing protease and phosphatase inhibitors and quantified using a BCA protein assay kit. (EC0001, SparkJade, China). The protein samples were treated with 10% SDS-PAGE and transferred to PVDF membrane. The nonspecific binding sites in the transfer membrane were blocked with 5% skim milk at room temperature for 2 h. Imprinted membranes were incubated with primary antibodies: RORγt protein antibody (1:2000, CST, 4967), Foxp3 protein antibody (1:1000, SANTA, sc28343), GPR43 (1:1000, Proteintech, 19,952-1-AP), NLRP3 (1:1000, Cell Signaling Technology, 15101S), Caspase-1 (1:1000, ABclonal, A0964), IL-1β (1:2000, Bioss, bs-0812R). 4 °C overnight. The membrane was incubated with HRP-labeled secondary antibody (1:2000) at room temperature for 1 h. After that, the bands were washed in TBST and detected by ELC ultra-sensitive chemiluminescence kit (ED0015-B, Sparkjade, China). The bands were quantified and analyzed by ImageJ.

In this study, β-actin and GAPDH were chosen as internal reference genes/proteins because they meet essential standards. β-actin is crucial for the cytoskeleton and cell functions, while GAPDH is vital for glycolysis and energy metabolism. Both are consistently expressed in various lung cells and remain stable under experimental conditions. The main factors in the obese asthma model are high-fat diet-induced obesity and OVA-sensitized asthma. The treatments did not significantly impact β-actin and GAPDH expression. While a high-fat diet altered systemic metabolism, GAPDH is basic metabolic function in lung tissue remained stable. OVA-induced lung inflammation affects inflammatory pathways but not β-actin or GAPDH. Expression levels were consistent across the normal, model, and drug intervention groups, making them suitable for detecting lung inflammation indicators.

### Reverse transcription-polymerase chain reaction

According to the requirements of the reverse transcription kit, the total RNA was extracted, and the template cDNA was synthesized by reverse transcription. The SYBR method was used for fluorescence quantitative PCR, and the real-time PCR instrument was used for PCR detection. The data were calculated by 2^−△△CT^ method with β-actin and GAPDH genes as internal reference. Each specimen was repeated three times, and each experiment was repeated three times. Primer sequence (Table 2).

**Table 2:** List of primers for real-time PCR.

Gene name	Primer sequence	
IL-1β	Forward	5′- TGCCACCTTTTGACAGTGATG -3’
	Reverse	5′- ATGTGCTGCTGCGAGATTTG -3’
NLRP-3	Forward	5′- TCTGCACCCGGACTGTAAAC -3’
	Reverse	5′- CATTGTTGCCCAGGTTCAGC -3’
FFAR2 (GPR43)	Forward	5′- GACAGGCTTCGGCTTCTACA -3’
	Reverse	5′- ACTGAACGATGATGACGATGGT -3’
Caspase-1	Forward	5′- ACATCTTTCTCCGAGGGTTGG -3’
	Reverse	5′- GGCAGGCAGCAAATTCTTTCA -3’
Rorγt	Forward	5′- GCTCCATATTTGACTTTTCCCACT -3’
	Reverse	5′- GATGTTCCACTCTCCTCTTCTCTTG -3’
Foxp3	Forward	5′- TACACCCAGGAAAGACAGCAAC -3’
	Reverse	5′- AGACTCCATTTGCCAGCAGT -3’
GAPDH	Forward	5′- GACATGCCGCCTGGAGAAAC -3’
	Reverse	5′- AGCCCAGGATGCCCTTTAGT -3’
β-actin	Forward	5′- GGCTGTATTCCCCTCCATCG -3’
	Reverse	5′- CCAGTTGGTAACAATGCCATGT -3’

### 16S *r*RNA gene sequencing and bioinformatics analysis

After the last administration, each group was fasted for 12 h, and 1–2 fresh feces in the colon were collected and transferred to −80 °C for further use after liquid nitrogen quick freezing. The genomic DNA of the samples was extracted by CTAB or SDS method, and then the purity and concentration of DNA were detected by agarose gel electrophoresis. An appropriate amount of sample DNA was taken in a centrifuge tube, and the sample was diluted with sterile water to 1 ng/μl. The diluted genomic DNA was used as a template for PCR according to the sequencing region (16S V3-V4), using Barcode-specific primers, New England Biolabs Phusion^®^ High-Fidelity PCR Master Mix with GC Buffer, and high-efficiency high-fidelity enzymes to ensure amplification efficiency and accuracy. PCR products were identified using 2% agarose gel electrophoresis. The suitable PCR products were purified with magnetic beads, quantified through enzyme labeling, and combined with equal amounts of samples based on their concentration. After full mixing, the PCR products were detected by 2% agarose gel electrophoresis, and the target bands were recovered by the gel recovery kit provided by Qiagen company. The was used to construct the library. The constructed library was quantified by Qubit and Q-PCR. After the library was qualified, NovaSeq 6000 was used for sequencing.

Each sample’s data was isolated from offline data based on the Barcode and PCR primer sequences, which were then removed. Fastp (v0.22.0) was used to filter the original reads for high quality by automatically detecting and removing joint sequences, eliminating reads with 15 or more N bases, discarding reads with over 50% low-quality bases (mass value ≤20), removing four bases with an average mass below 20 in a window, deleting polyG tails, and excluding reads shorter than 150 bp. High-quality paired-end reads were processed with FLASH (v1.2.11) to obtain Clean Tags. These Tags were compared to a species annotation database using vsearch (v2.22.1) to identify and remove chimera sequences, resulting in the final Effective Tags. The ASV sequences were annotated using Mothur (v1.48) and SILVA138.1 methods. Species annotation was conducted with the SSUrRNA database (28) using a threshold of 0.8 to 1 for taxonomic classification at levels from phylum to species. Community composition for each sample was then assessed. MAFFT (v7.520) was employed for multiple sequence alignment to determine the phylogenetic relationships of ASV representative sequences. Data were homogenized using the smallest sample size as a standard, forming the basis for subsequent Alpha and Beta diversity analyses.

### Fecal short-chain fatty acids (SCFAs)

To measure fecal SCFAs concentrations, gas chromatography/mass spectrometry was employed. 20 mg of fecal samples were placed in a 2 mL microcentrifuge tube, to which 1 mL of phosphoric acid (0.5% v/v) and a small steel ball were added. The combination was pulverized three times for 10 s each, then subjected to vortexing for 10 min and ultrasonication for 5 min. The sample was spun at 12,000 rpm for 10 min at 4 °C, and 0.1 mL of the supernatant was moved to a 1.5 mL centrifuge tube. Then, 0.5 mL of methyl tert-butyl ether (MTBE) solution was added to the tube. The blend was stirred with a vortex for 3 min, then subjected to ultrasonication for 5 min, spun at 12,000 rpm for 10 min at 4 °C, and analyzed using gas chromatography/mass spectrometry (GC/MS) on an Agilent7890B-7000D system (Agilent Technologies, CA, USA). Acetate, propionate, and butyrate were quantified using pure standards diluted in diethyl ether.

### Statistical analysis

Data from the experiments came from at least three replicates and were shown as mean ± SEM. All analyses were carried out using GraphPad Prism 8.0. Regarding self-control data before and after, the paired *t*-test is applied if the variable difference follows a normal distribution. The Student’s *t*-test was employed for comparing two groups, while one-way ANOVA was used for comparing multiple groups. *p* < 0.05 was considered statistically significant. All *p*-values are shown in the figure (*p < 0.05, **p < 0.01; as compared to the n group. #*p* < 0.05, ##*p* < 0.01; as compared to the fa group).

### Key instructions for experimental design

In this study, the initial sample size for each group was established at *n* = 10 to ensure the statistical robustness of the experimental outcomes. During the subsequent data collection phase, for laboratory assays such as ELISA (cytokine detection) and qPCR (gene expression analysis), a “random sampling detection” approach was employed. Specifically, samples from 5 mice were randomly selected from each group for testing, while the remaining 5 samples were preserved for future use, such as repeated verification or supplementary testing. The random number table method was utilized to conduct the sampling process, thereby ensuring the representativeness of the test samples.

The experiment adhered rigorously to the principle of randomization. Both the initial and secondary animal groupings, following successful model establishment, as well as the sampling of test specimens, utilized a random number table method to eliminate potential selection bias. A double-blind methodology was employed throughout the experimental process: (1) Personnel responsible for drug administration were aware only of the group numbers and were blinded to the specific treatments assigned to each group (i.e., normal control, model, or drug intervention). (2) Individuals involved in sample collection, laboratory testing (including ELISA, qPCR, and pathological section analysis), and data statistical analysis were also blinded to the group assignments and treatment protocols until the completion of data analysis. This approach was implemented to minimize the influence of subjective bias on the experimental outcomes.

## Results

### Network pharmacology prediction of XBCQD for obese asthma

**Figure 3:**
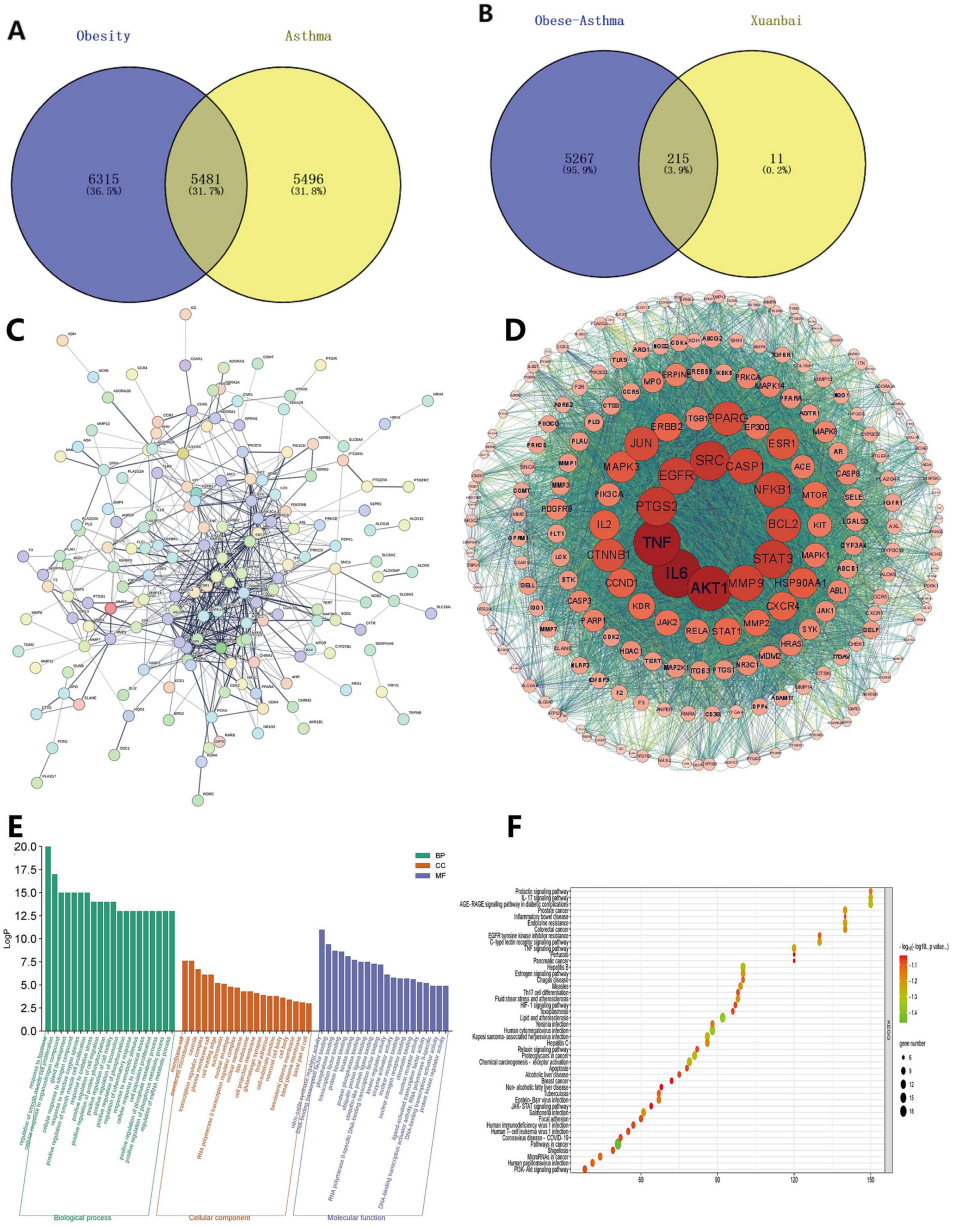
The target characteristics of XBCQD in the treatment of obese asthma were determined by network pharmacology. **(A,B)** Enter the screened targets and disease targets into Venn diagram software to identify 215 common targets, which were used as drug targets. Pathway enrichment analysis was carried out next. **(C,D)** To create the PPI network, frequent targets and diseases were entered into the String. **(E,F)** GO enrichment and KEGG analysis.

In order to explore the mechanism of XBCQD in the treatment of obese asthma, network pharmacology was used to analyze its potential targets. The results showed that 19 main active components of Xuanbai Chengqi Decoction were identified and screened, including 5 rhubarb, 4 trichosanthis, 10 almonds (Table 3), 226 drug targets and 5,481 disease targets (Figure 3A). Relevant literature reports and our previous studies on the identification and quantitative analysis of the main active components of XBCQD also support this result (29, 30). The intersection of XBCQD drug targets and obesity-asthma disease targets was obtained, and 215 potential target genes were obtained and the active components in the drug and the drug-disease common target venny diagram were drawn (Figure 3B). The 215 common targets were input into the STRING database to construct a ‘drug-disease’ visual PPI network (Figure 3C). The PPI network has 105 nodes and 602 edges, with an average node degree of 11.5. Import these results into Cytoscape 3.8.0 (Figure 3D). The first 12 targets were selected, followed by IL-6, TNF, AKT1, SRC, PTGS2, STAT3, EGFR, MMP9, NFKB1, CASP1, BCL2, JUN, PPARG, CTNNB1, ESR1, MAPK3, HSP90AA1, IL2, CCND1, CXCR4, ERBB2, STAT1. These targets may be the key targets of Xuanbai Chengqi Decoction in the treatment of obesity-related asthma.

**Table 3:** The main active ingredients of XBCQD.

Source	Serial number	Active ingredient	Chemical formula	CAS no.	Oral bioavailability %	Drug-likeness
Da Huang	MOL002235	EUPATIN	C18H16O8	19587-65-6	50.80	0.41
Da Huang	MOL002268	Rhein	C15H8O6	478-43-3	47.07	0.28
Da Huang	MOL002281	Toralactone	C15H12O5	41743-74-2	46.46	0.24
Da Huang	MOL000471	Aloe-emodin	C15H10O5	481-72-1	83.38	0.24
Da Huang/Ku Xing Ren	MOL000096	Catechin	C15H14O6	154-23-4	49.68	0.24
Gua Lou	MOL002881	Diosmetin	C16H12O6	520-34-3	31.14	0.27
Gua Lou	MOL005530	Hydroxygenkwanin	C16H12O6	20243-59-8	36.47	0.27
Gua Lou	MOL007172	7-Oxodihydrokarounidiol	C30H48O3	143183-47-5	36.85	0.75
Gua Lou	MOL007179	Ethyl linolenate	C20H34O2	1191-41-9	46.10	0.20
Ku Xing Ren	MOL010921	Estrone	C18H22O2	53-16-7	55.38	0.78
Ku Xing Ren	MOL010922	Diisooctyl succinate	C20H38O4	28880-24-2	31.62	0.23
Ku Xing Ren	MOL002311	Glycyrol	C21H18O6	23013-84-5	90.78	0.67
Ku Xing Ren	MOL004841	Licochalcone B	C16H14O5	58749-23-8	76.76	0.19
Ku Xing Ren	MOL004908	Glabridin	C20H20O4	59870-68-7	53.25	0.47
Ku Xing Ren	MOL005017	Phaseol	C20H16O5	88478-02-8	78.77	0.58
Ku Xing Ren	MOL007207	Machiline	C17H19NO3	2196-60-3	79.64	0.24
Ku Xing Ren	MOL012922	l-SPD	C19H21NO4	16562-13-3	87.35	0.54
Ku Xing Ren	MOL003410	Jujubogenin	C30H48O4	54815-36-0	66.95	0.62

The R language package was used to analyze GO and KEGG functional enrichment, and the *p*-value of the common goal was <0.05. The histogram and bubble diagram were used to visualize the first 20 rich factor results and the first 45 paths. BP enrichment mainly includes hormone response, regulation of smooth muscle cell factor proliferation, cell response to organic nitrogen compounds, gland development, cell response to nitrogen compounds, and response to reactive oxygen species. CC enrichment mainly includes membrane rafts, membrane microdomains, cell membrane pits, transcriptional regulatory complexes, plasma membrane rafts, etc. MF enrichment mainly included nitric oxide synthase regulator activity, DNA binding transporter binding, phosphatase binding, transcription factor binding, and protein kinase binding (Figure 3E). The KEGG pathway related to obese asthma mainly involves the IL-17 signaling pathway, the AGE-RAGE signaling pathway, the TNF signaling pathway, Th17 cell differentiation, and so on (Figure 3F).

### XBCQD improved the respiratory function of asthmatic mice

Following the replication and categorization of the obese asthma model mice as per the previously established methodology, we assessed the fundamental lung function of each group utilizing a non-invasive lung function meter. The findings indicated that the Specific Airway Resistance (SAR) in each group escalated in a dose-dependent manner with increasing Methacholine concentrations. In comparison to the *n* group, the f and fa groups exhibited a significant increase in SAR under varying Methacholine concentrations, indicative of pronounced airway hyperresponsiveness. Notably, the f group demonstrated elevated SAR levels even at a Methacholine concentration of 0 mg/mL. As the Methacholine concentration increased, the progression of airway resistance in this group was relatively gradual. This phenomenon may be attributed to the accumulation of adipose tissue in the thoracic and abdominal regions, which mechanically compresses the thoracic cavity, restricts diaphragmatic movement, elevates pleural pressure, reduces lung compliance, and consequently increases airway resistance (31). Compared with the fa group, XBCQD and montelukast sodium alleviated airway hyperresponsiveness after intervention (Figure 2D).

### Effects of XBCQD on lung histopathology and IgE in alveolar lavage fluid of obese asthmatic mice

Hematoxylin and eosin (HE) staining was employed to evaluate the effects of XBCQD on the lung tissue of obese asthmatic subjects. The findings indicated that the airway epithelium in group n was intact and well-organized, with normal bronchial architecture, no significant infiltration or aggregation of inflammatory cells, and an absence of inflammatory secretions in the bronchial and alveolar spaces. In contrast, the fa group exhibited extensive inflammatory cell infiltration and damage to the alveolar walls surrounding the bronchi. Group f showed no significant damage to lung tissue. Following XBCQD intervention, the xl, xm, and xh groups demonstrated varying degrees of improvement in the infiltration of inflammatory cells around the trachea and alveoli in obese asthmatic mice. These results suggest that XBCQD exerts a beneficial effect on lung histopathology (Figure 2B).

ELISA results showed that the content of IgE in the alveolar lavage fluid of mice in the fa group increased significantly (*p* < 0.01). The difference between the f group and n group was small, and the difference between the f group and fa group was significant (*p* < 0.01). After treatment with Xuanbai Chengqi Decoction, the alveolar lavage fluid of mice in each group decreased significantly (*p* < 0.01), among which the xh and xl groups had the best effect (Figure 2C).

### The effects of XBCQD on body weight, blood glucose, blood lipid, and fat index in obese asthmatic mice

In order to study the effect of XBCQD on obesity in obese asthma, there was no significant difference in body weight of mice before modeling (*p* > 0.05). After 11 weeks of high-fat diet feeding, the weight of mice fed with high-fat diet increased significantly (*p* < 0.01), and the weight gain caused by the high-fat diet was slowed down by intragastric administration of Xuanbai Chengqi Decoction (*p* < 0.05) (Figure 2E).

Compared with the n group, the levels of TC and TG in other groups were significantly increased, and the levels of TC and TG in the serum of mice in the f and fa group were significantly increased (*p* < 0.01). Compared with the fa group, serum TC and TG in xl and xh groups were significantly decreased (*p* < 0.05) (Figures 2F,G).

Compared with normal mice, the fat index of model mice in the f and fa groups increased significantly (*p* < 0.01). Compared with the fa group, the fat index of XBCQD and montelukast sodium decreased significantly after treatment (*p* < 0.01). Among them, the fat index of the xh group decreased most significantly, followed by the xl group. The experimental results suggest that both XBCQD and montelukast sodium can reduce the content of abdominal white fat in mice (Figures 2H, I).

OGTT results indicated that the blood glucose levels in the high-fat diet mice were measured at 0, 15, 30, 60, and 90 min, with a peak observed at approximately 15 min. The baseline blood glucose levels in the fa and f groups were significantly elevated compared to the n group mice. The area under the curve (AUC) analysis corroborated that a high-fat diet led to increased blood glucose levels and impaired glucose tolerance in mice (Figures 2J,K). Furthermore, treatment with XBCQD ameliorated the impaired glucose tolerance observed in HFD-fed mice, with a notable reduction in blood glucose levels, particularly in the xh group (Figure 2K).

### XBCQD regulates GPR43/NLRP3 inflammasome signaling pathway

**Figure 4:**
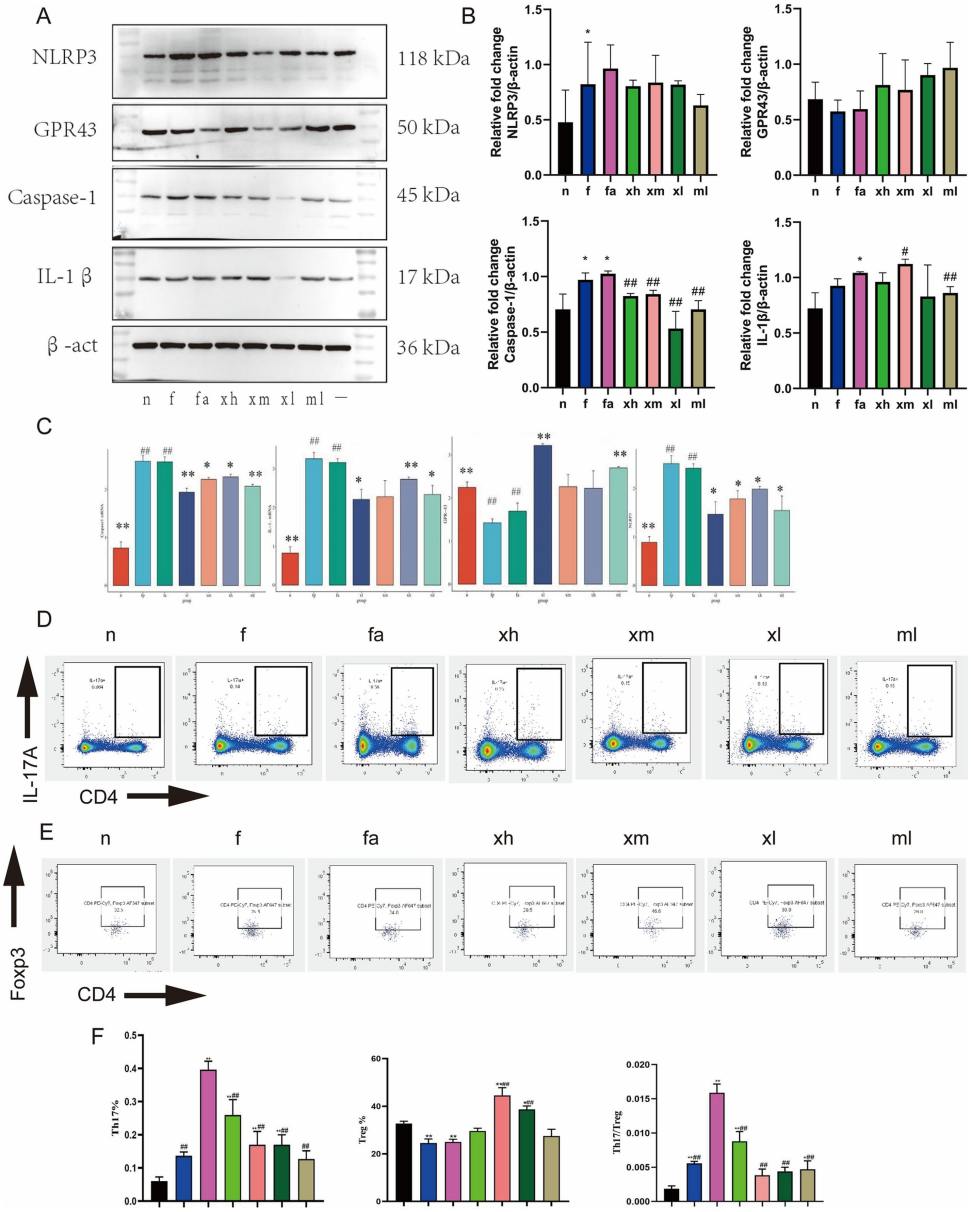
XBCQD regulates Th17/Treg immune imbalance through GPR43/NLRP3. **(A,B)** The protein expression of NLRP3, GPR43, Caspase-1, and IL-1β in lung tissue after XBCQD treatment (*n* = 5). **(C)** The mRNA expression of NLRP3, GPR43, Caspase-1, and IL-1β in lung tissue after XBCQD treatment (*n* = 5). **(D–F)** Flow cytometry of Th17 cells in lung tissue. The representative plots of Th17 cells gated by CD3^+^CD4^+^IL17^+^; The representative plots of Treg cells gated by CD3^+^CD4^+^CD25^+^Foxp3^+^ (*n* = 5) (*p < 0.05, **p < 0.01; as compared to the *n* group. #*p* < 0.05, ##*p* < 0.01; as compared to the fa group).

To explore whether Xuanbai Chengqi Decoction plays a pharmacological role through the GPR43/NLRP3 signaling pathway. RT-qPCR and Western Blot analyses were conducted on mouse lung tissue, revealing that GPR43 mRNA expression in the fa group was notably reduced compared to the n group following a high-fat diet and OVA stimulation (*p* < 0.01), while the mRNA expression levels of NLRP3, Caspase-1, and IL-1β were significantly up-regulated (*p* < 0.01). After treatment with Xuanbai Chengqi Decoction, xl, xm, and xh significantly up-regulated the expression of GPR43 mRNA (*p* < 0.01), and inhibited the expression of NLRP3, Caspase-1, and IL-1β (*p* < 0.01) (Figures 4A–C). Western Blot showed that stimulation increased the protein expression of Caspase-1 and IL-1β in the lungs of obese asthmatic mice, but these increases were significantly inhibited in the xl group. It is worth noting that the protein expression of GPR43 increased to varying degrees after treatment (Figures 4A–C). These findings suggest that XBCQD inhibits the activation of the NLRP3 inflammasome by activating GPR43.

### XBCQD corrects Th17/Treg cell imbalance and reduces related cytokines

Th17 and Treg cells are critical in modulating airway inflammation in patients with asthma, acting to either promote or inhibit this condition. Observations of differential symptom improvement between obese asthma model mice and those in the treatment group led us to employ flow cytometry to quantify the percentages of Th17 and Treg cells within spleen tissue. IL-17A was utilized as a marker to indicate the proportion of Th17 cells. Notably, the proportion of Th17 cells in the spleen tissue of mice in the fa group (0.4 ± 0.03%) was significantly elevated compared to the n group (0.06 ± 0.01%). Following pharmacological intervention, a significant reduction in the proportion of Th17 cells was observed (*p* < 0.01), with the most pronounced decrease occurring in the ml group, followed by the xm and xl groups. The proportion of Treg cells was assessed using Foxp3 as a marker. In comparison to the n group, the fa group exhibited a significant reduction in the proportion of Treg cells post-modeling (*p* < 0.01) (Figures 4E,F). Following treatment, there was a significant increase in the proportion of Treg cells in the xm and xl groups (*p* < 0.01), while the xh and ml groups showed no significant change (Figures 4E,F). Our study showed that after high-fat diet combined with OVA stimulation, the ratio of Th17/Treg in fa group mice increased significantly (*p* < 0.01) (Figures 4D,F), After drug treatment, the ratio of Th17/Treg in mice decreased (*p* < 0.01), and the decrease in xm group was the most obvious (Figures 4D,F). It is suggested that after modeling, obese asthma can lead to Th17/Treg immune imbalance. XBCQD treatment significantly inhibits Th17 cell differentiation and promotes the development of Treg cell differentiation, and improves immune imbalance in obese asthmatic mice by regulating the proportion of Th17/Treg cells.

**Figure 5:**
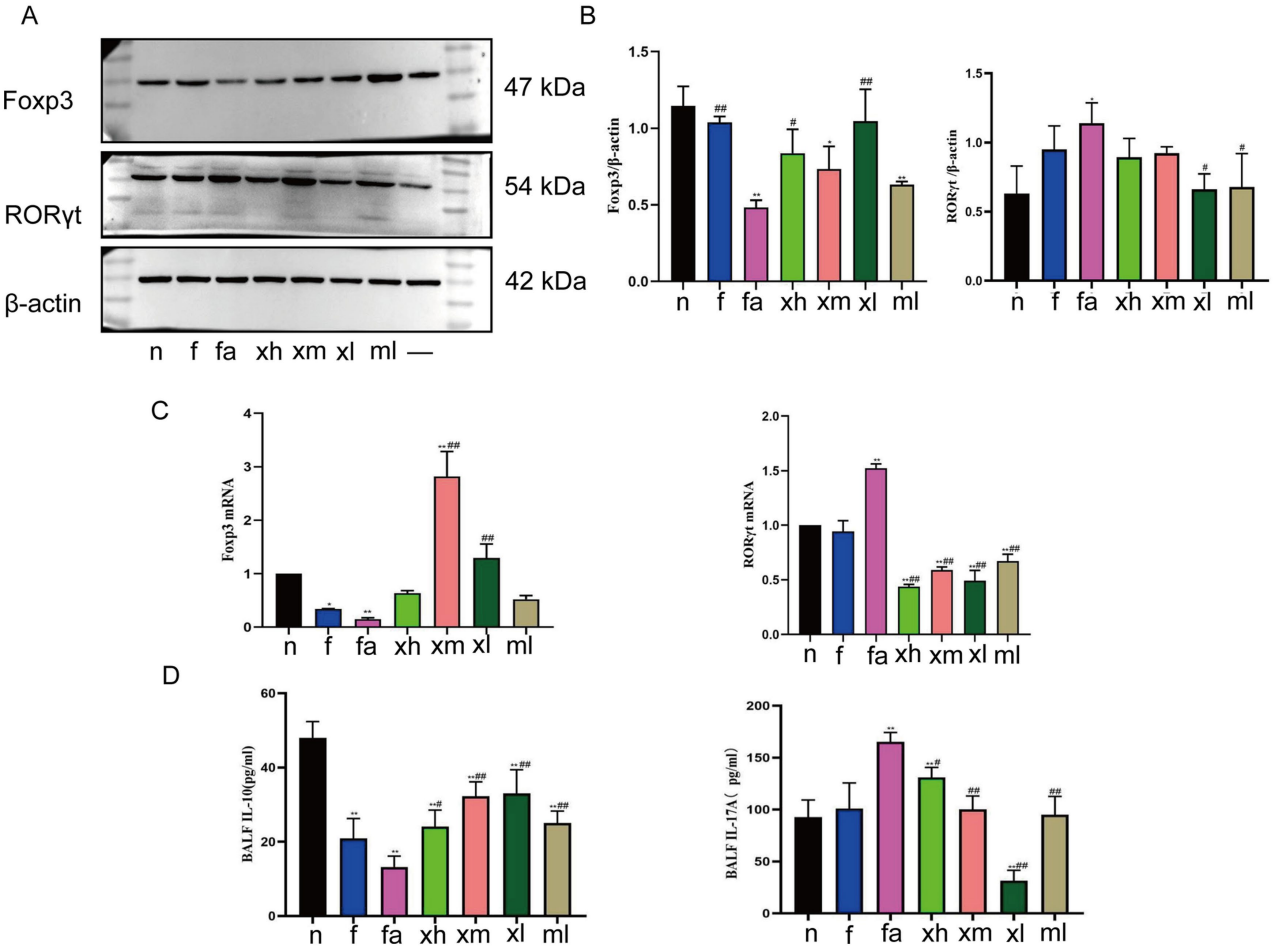
XBCQD regulates Th17/Treg-related cytokines. **(A,B)** The protein expression of Foxp3 and RORγt in lung tissue after XBCQD treatment (*n* = 5). **(C)** The mRNA expression of Foxp3 and RORγt in lung tissue after XBCQD treatment (*n* = 5). **(D)** The expression of IL-10 and IL-17A in BALF after XBCQD treatment (*n* = 5). (*p < 0.05, **p < 0.01; as compared to the *n* group. #*p* < 0.05, ##*p* < 0.01; as compared to the fa group).

Subsequently, we used RT-qPCR, ELISA, and Western Blot to detect and verify Th17 and Treg-related cytokines in mouse lung tissue. RORγt is a specific transcription factor of Th17. Compared with the n group, the expression of RORγt mRNA in the fa group was significantly increased (*p* < 0.01) (Figure 5C). After treatment, the expression level of RORγt mRNA in mice was significantly decreased (*p* < 0.01), and the decrease in the xh and xl group was the most obvious (Figure 5C). Western Blot results also showed that the expression of RORγt protein in lung tissue of mice stimulated by high-fat diet and OVA tended to increase (Figures 5A,B). After the intervention of Xuanbai Chengqi Decoction and western medicine, it showed a decreasing trend, among which the xl group was the most obvious (*p* < 0.05) (Figures 5A,B). ELISA results showed that the expression level of IL-17A in the alveolar lavage fluid of mice in the fa group was significantly increased after high-fat diet and OVA stimulation (*p* < 0.01) (Figure 5D). After the intervention of Xuanbai Chengqi Decoction, the expression level of IL-17A in the xm group, the xl group, and the ml group was significantly lower than that in the fa group (*p* < 0.01) (Figure 5D).

Forkhead box P3 (Foxp3) is a transcription factor specific to regulatory T (Treg) cells. Reverse transcription quantitative polymerase chain reaction (RT-qPCR) was employed to quantify the expression of Foxp3 in lung tissue samples. Relative to group n, the expression levels of Foxp3 mRNA in the lung tissues of groups f and fa were significantly reduced, with the fa group exhibiting particularly low levels of expression (*p* < 0.01) (Figure 5C). After treatment, the expression level of Foxp3 mRNA increased, and xm and xl group increased significantly (*p* < 0.01) (Figure 5C). Western Blot results also showed that the expression of Foxp3 protein in lung tissue of mice in fa group was decreased (*p* < 0.01) (Figures 5A,B). The expression of Foxp3 protein was restored to a certain extent after treatment with Xuanbai Chengqi Decoction, and the expression of Foxp3 protein in the xl group was improved most significantly (*p* < 0.01) (Figures 5A,B). IL-10 is a representative cytokine of Treg cells. Compared with group n, the expression of IL-10 in the alveolar lavage fluid of mice in group f and group fa was significantly decreased (*p* < 0.01) (Figure 5D). The expression level of the fa group was the lowest. After the intervention of Xuanbai Chengqi Decoction and montelukast sodium, the expression of IL-10 increased, and the xm group, xl group, and ml group improved significantly (*p* < 0.01) (Figure 5D). The experimental results suggest that Xuanbai Chengqi Decoction can increase the expression of Foxp3 and IL-10, inhibit the expression of RORγt and IL-17A, increase the level of Treg, and reduce the proportion of Th17 cells to restore Th17/Treg immune imbalance.

### XBCQD alters gut microbiota diversity in obese asthmatic mice

**Figure 6:**
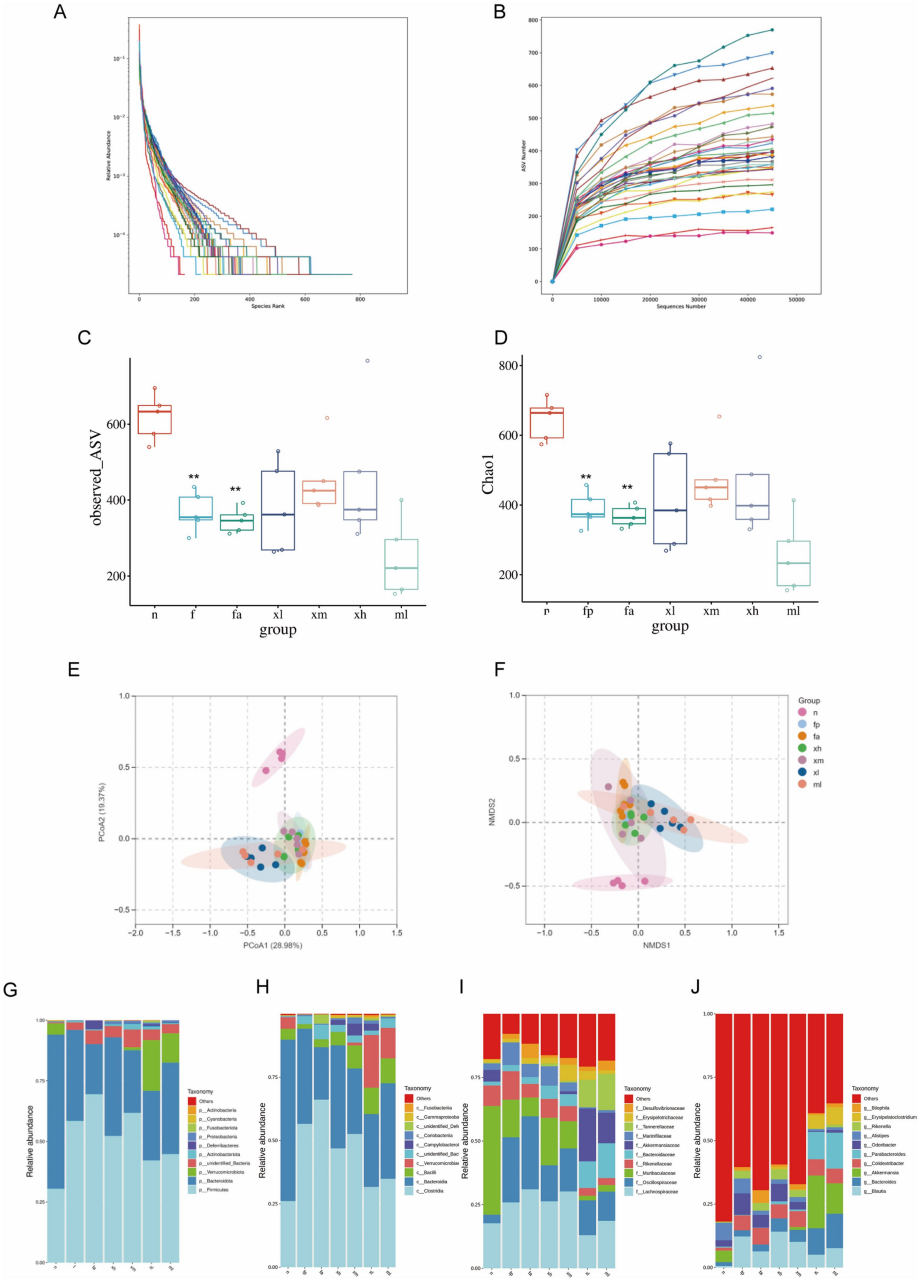
Changes of intestinal microflora diversity in obese asthmatic mice after XBCQD treatment. **(A)** Rarefaction curves (*n* = 5). **(B)** Curve showing rank abundance (*n* = 5). **(C,D)** Observed species index and Chao1 index (*n* = 5). **(E,F)** PCoA2 and NMDS2 (*n* = 5). **(G)** At the phylum level, the relative abundance of microbial species is shown (*n* = 5). **(H)** At the class level, the relative abundance of microbial species is shown (*n* = 5). **(I)** At the family level, the relative abundance of microbial species is shown (*n* = 5). **(J)** Relative abundance of microbial species at the genus level (*n* = 5) (**p < 0.01; as compared to the *n* group).

Most intestinal microbes can be classified at the genus level. The slope of the rarefaction curve approaches saturation, indicating that the sequencing depth is adequate (Figure 6A). The curve depicting species abundance declines steadily before plateauing (Figure 6B). The extensive range of the horizontal axis suggests a rich and even distribution of species samples. Alpha diversity analysis revealed that a high-fat diet combined with OVA stimulation significantly reduced the diversity of the intestinal microbiota (Figure 6C). Compared to group n, the Chao1 index and ASV diversity index of f and fa groups decreased significantly (*p* < 0.01) (Figure 6D). Following XBCQD treatment, the diversity of the intestinal microbiota showed varying degrees of recovery (Figure 6D). Notably, the intestinal microbiota of mice treated with montelukast sodium continued to decline, exhibiting lower richness and diversity than f and fa groups, suggesting that montelukast sodium may inhibit the richness and diversity of the intestinal microbiota (Figure 6D). PCoA2 and NMDS2 were performed on the basis of ASV for β diversity analysis. The projection distances of the samples in the n group and the other groups in the vertical coordinate system were significantly different, indicating that the intestinal flora structure would be affected by high-fat diet and asthma (Figures 6E,F).

To comprehend how XBCQD influences the intestinal microbial makeup in obese asthmatic mice, we examined the microbial composition across phylum, class, family, and genus levels. At the gate level, *Bacteroidota* accounted for the highest proportion (63%) in the n group, followed by *Firmicutes* (30%); obese asthma stimulation resulted in a significant increase in *Firmicutes* (69%) and a significant decrease in *Bacteroidota* (20%). The imbalance between the two was alleviated to some extent after treatment (Figure 6G). At the class level, *Coriobacteriia* was enriched in the n group, while the fa group decreased most significantly. XBCQD and montelukast sodium treatment improved the proportion of *Coriobacteriia* in the flora; the fa group was enriched with a large number of *Clostridia* (66%), which was reversed after treatment (Figure 6H). At the family level, *Tannerellaceae* dominated in the n group, obese asthma increased *Lachnospiraceae*, and this trend was alleviated after treatment (Figure 6I). At the genus level, *Alistipes* was more abundant in the n group, while *Colidextribacter* and *Blautia* were more abundant in the fa group. After treatment, there was no significant increase in the level of *Alistipes*, but the level of *Akkermansia* increased significantly, especially in the xm, xl, and ml groups (Figure 6J).

### The effect of XBCQD on specific intestinal microorganisms and metabolites, SCFAs

**Figure 7:**
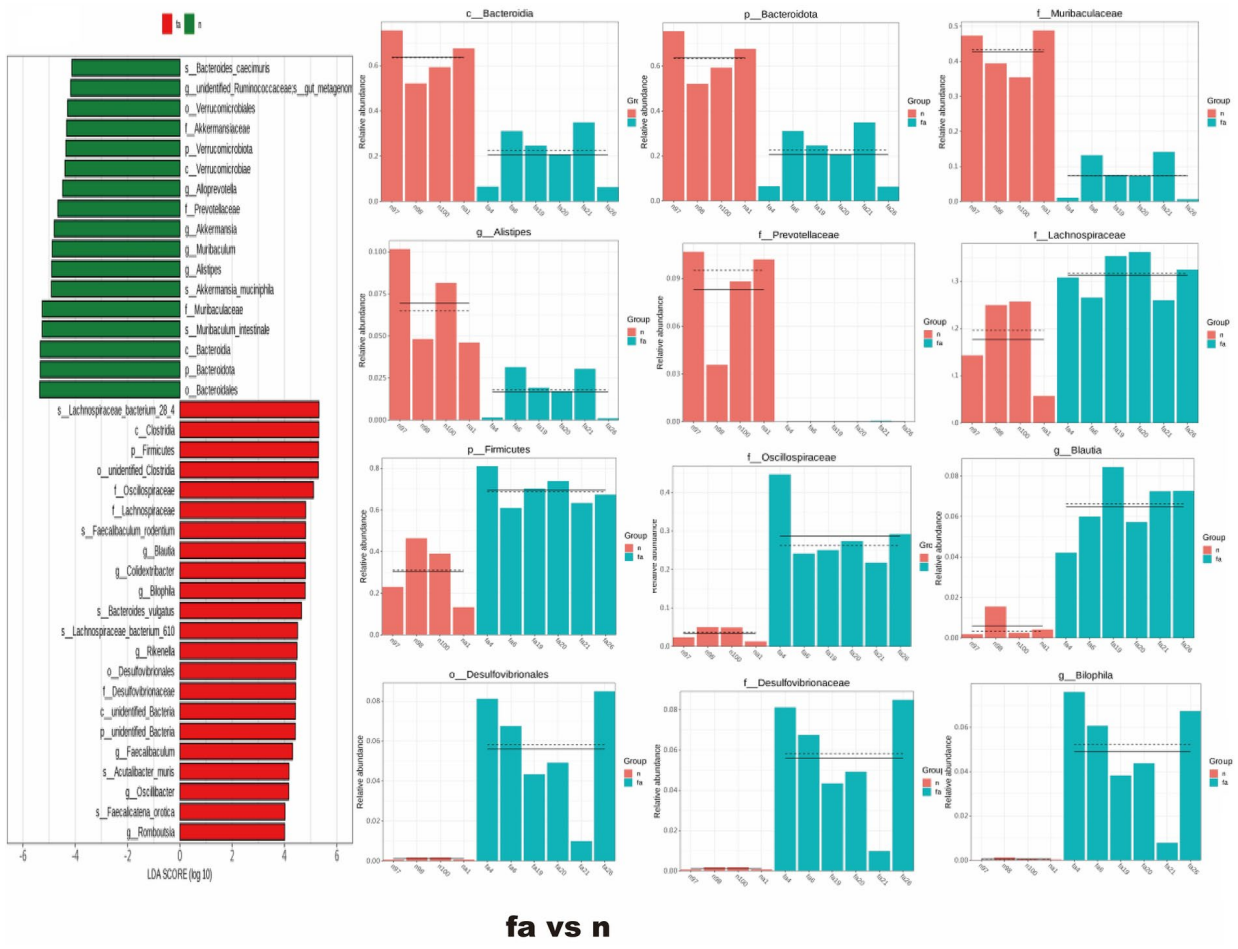
Changes of intestinal microbial diversity in obese asthmatic mice and normal mice. The results of differential flora between the fa group and n group based on LEfSe analysis (*n* = 5).

**Figure 8:**
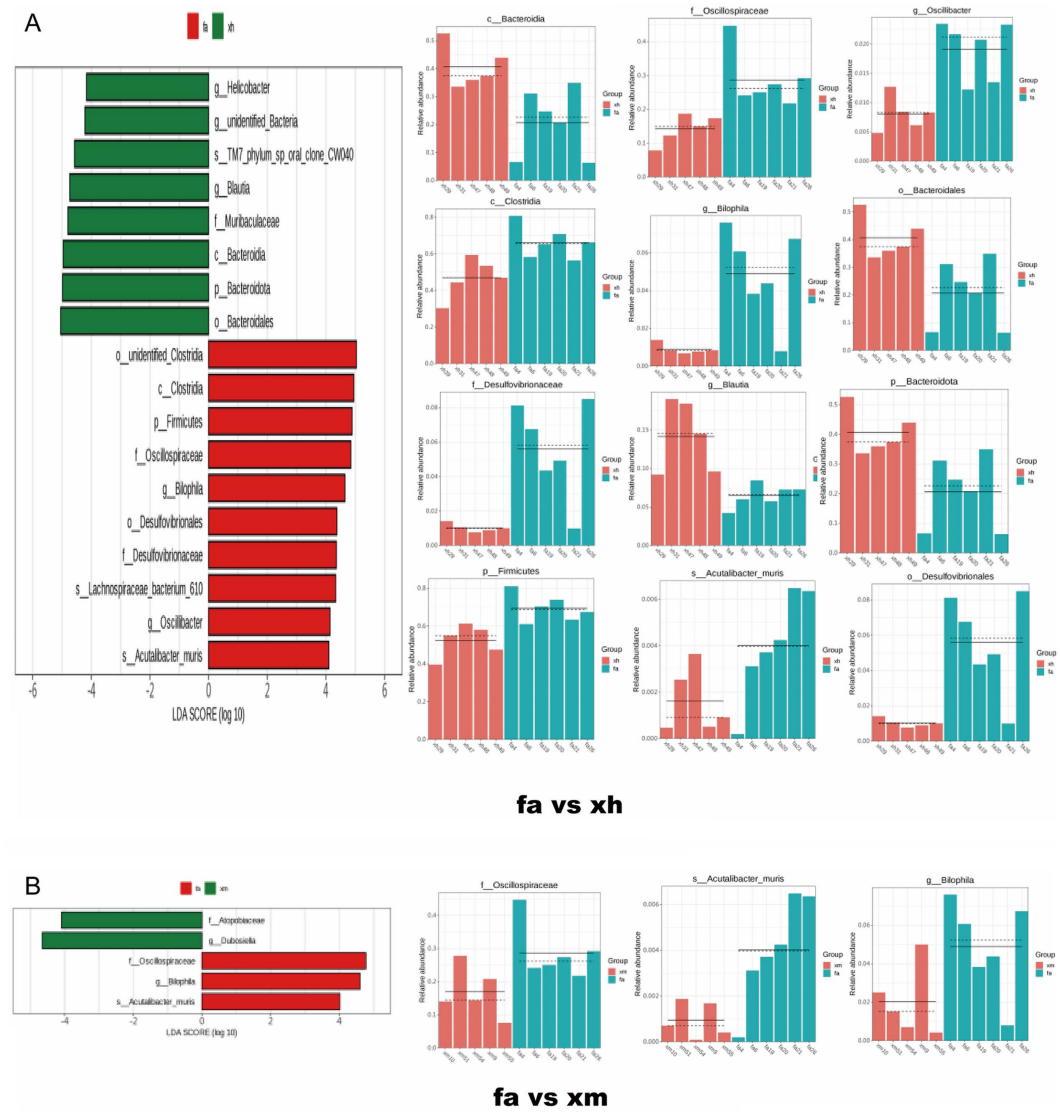
The changes of intestinal microbial diversity in XBCQD-treated mice and obese asthmatic mice. **(A)** The results of differential flora between the FA group and XH group based on LEfSe analysis (*n* = 5). **(B)** The results of differential flora between the FA group and XM group based on LEfSe analysis (*n* = 5).

**Figure 9:**
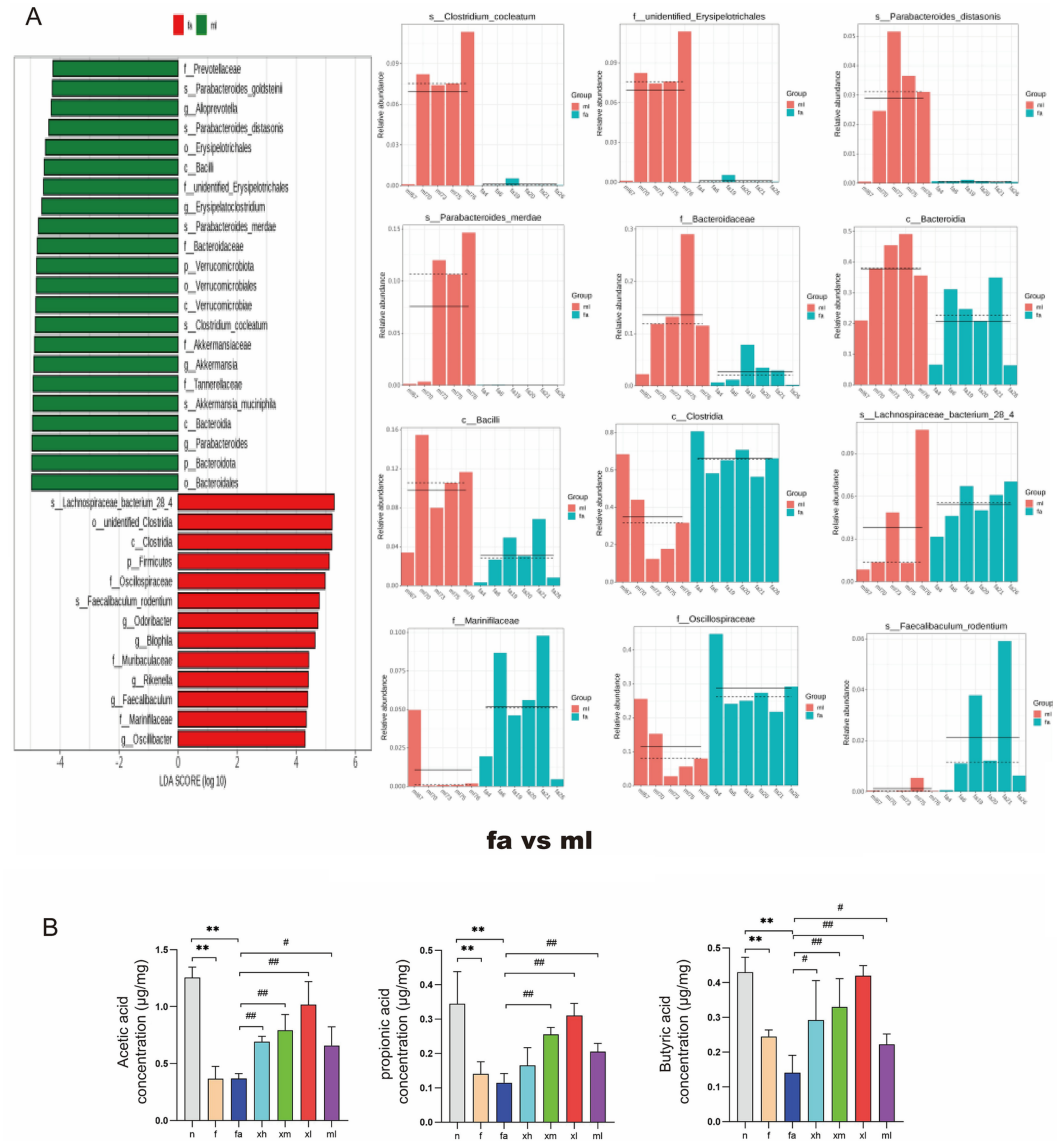
The changes of intestinal microbial diversity in montelukast sodium-treated mice and obese asthmatic mice; changes of SCFAs in intestinal contents of mice. **(A)** The results of differential flora between the FA group and ML group based on LEfSe analysis (*n* = 5). **(B)** SCFAs levels in intestinal contents (acetic acid, propionic acid, butyric acid) (*n* = 5) (**p < 0.01; as compared to the n group. ^#^*p* < 0.05, ^##^*p* < 0.01; as compared to the fa group).

Later, to assess the bacterial index across different groups, LEfSe analysis was performed to identify species with significant differences in abundance between the two groups. In the fa group, 22 bacterial taxa were significantly enriched compared to the n group (Figure 7), including *Lachnospiraceae_bacterium_28_4*, *Clostridia*, *Firmicutes*, *Oscillospiraceae*, *Lachnospiraceae*, *Desulfovibrionales*, among others. Conversely, the abundance of 8 bacterial taxa was significantly restored in the xh group compared to the fa group (Figure 8A), such as *Bacteroidales*, *Bacteroidota*, *Muribaculaceae*, *Blautia*, and others. Compared to the fa group, only two bacterial taxa were significantly enriched in the xm group (Figure 8B), including *Bacteroidales*, *Bacteroidota*, *Muribaculaceae*, and Blautia; whereas, only two types of bacteria, *Dubosiella* and *Atopobiaceae*, were significantly enriched in the xm group compared to the fa group (Figure 8B). The xl group showed a significant enrichment of 23 bacterial types relative to the fa group, including *Akkermansia_muciniphila*, *Akkermansia*, *Verrucomicrobiae*, *Akkermansiaceae*, *Verrucomicrobiota*, *Verrucomicrobiales*, *Parabacteroides*, *Tannerellaceae*, *Parabacteroides_merdae*, *Clostridium_cocleatum*, and *Bacteroidaceae*. Additionally, 22 bacterial types, such as *Bacteroidales*, *Bacteroidota*, *Parabacteroides*, *Bacteroidia*, *Akkermansia_muciniphila*, *Tannerellaceae*, *Akkermansia*, *Akkermansiaceae*, *Clostridium_cocleatum*, *Verrucomicrobiae*, and others, were significantly enriched in the ml group compared to the fa group (Figure 9A).

To explore the effect of XBCQD on SCFAs, the level of SCFAs in intestinal contents was determined by GC/MS. It was found that the levels of SCFAs in f group and fa group were significantly decreased (*p* < 0.01) (Figure 9B). Simultaneously, XBCQD treatment notably raised the levels of acetic acid, propionic acid, and butyric acid (*p* < 0.01), while montelukast sodium treatment notably elevated the concentration of propionic acid (*p* < 0.01) (Figure 9B). Our results showed that XBCQD increased the level of SCFAs in the colon. Intestinal microorganisms are an important aspect of the lung-gut axis. XBCQD may alleviate airway inflammation by regulating intestinal microorganisms and their metabolites, SCFAs.

### Correlation between intestinal flora and related indicators

**Figure 10:**
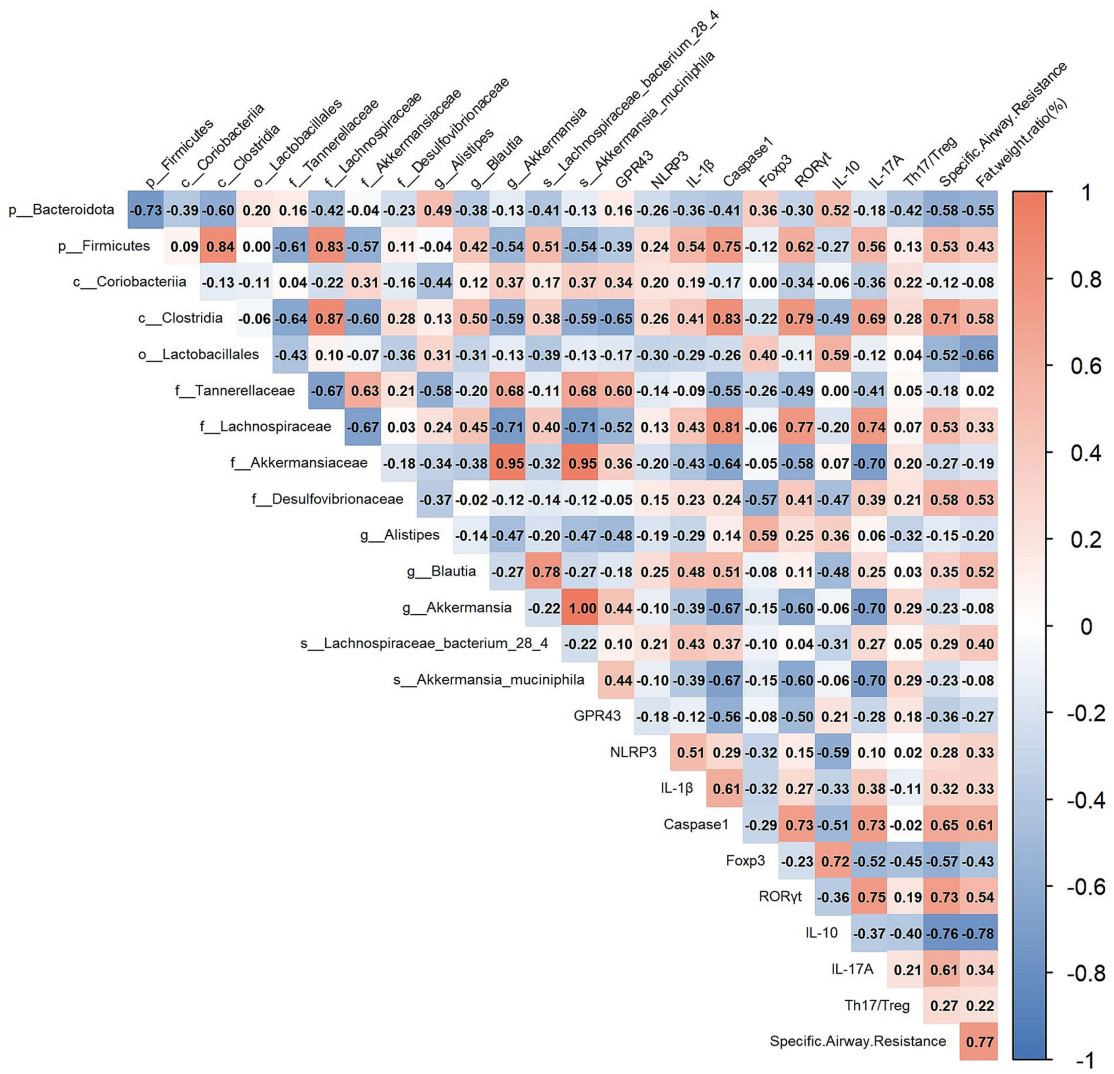
Intestinal microorganisms and related indicators. Pearson’s correlation analysis was performed on intestinal microflora abundance and related indicators.

To explore the relationship between intestinal flora and related indicators, Pearson’s correlation analysis was performed and a heat map of correlation coefficients was achieved. The findings indicated that *Bacteroidota*, *Lactobacillales*, *Tannerelaceae*, *Akkermansiaceae*, *Akkermansia*, *Akkermansia_muciniphila*, and *Alistipes* exhibited a positive correlation with the reduction of NLRP3, IL-1β, and Caspase1 levels. Additionally, *Bacteroidota* and *Alistipes* were positively associated with the restoration of the Th17/Treg ratio. Furthermore, *Bacteroidota*, *Lactobacillales*, *Akkermansiaceae*, *Alistipes*, and *Akkermansia_muciniphila* demonstrated a positive correlation with the enhancement of airway resistance. Lastly, *Bacteroidota*, *Lactobacillales*, *Akkermansiaceae*, *Akkermansia*, *Akkermansia_muciniphila*, and *Alistipes* were positively correlated with a reduction in obesity (Figure 10).

## Discussion

This study investigates the therapeutic effects and underlying molecular mechanisms of Xuanbai Chengqi Decoction (XBCQD) utilizing a murine model of obesity-induced asthma. The principal findings suggest that XBCQD potentially reinstates the Th17/Treg immune equilibrium by modulating the gut-lung axis, specifically through the gut microbiota-SCFAs/GPR43/NLRP3 signaling pathway. This modulation appears to ameliorate airway inflammation and metabolic dysregulation associated with obesity-related asthma.

In this study, 4-week-old female C57BL/6 J mice were utilized to establish a model of “high-fat diet-induced obesity combined with OVA-sensitized asthma” (32). The findings indicated that the body weight of mice in the f and fa groups exceeded that of the n group by more than 20%. Additionally, serum TC and TG levels were significantly elevated, and OGTT revealed impaired glucose tolerance, aligning with the fundamental characteristics of obesity and metabolic disorders. Mice in the fa group exhibited symptoms of cough and wheezing. Lung function tests demonstrated increased airway resistance, pronounced inflammatory cell infiltration around the bronchus, significantly elevated IgE levels in BALF, and increased collagen deposition and TGF-β expression in lung tissue. These findings corroborate previous reports that airway remodeling in the obese asthma model is more pronounced in female mice (33–35). The above data jointly verified the successful construction of the model, which not only simulated the metabolic phenotype of obesity but also reproduced the airway inflammation and remodeling characteristics of asthma, providing a reliable experimental basis for the subsequent evaluation of the therapeutic effect of XBCQD. Throughout the modeling period, with the exception of two mice that exhibited behavioral disorders attributed to the combined stress of a high-fat diet and OVA stimulation, the remaining mice survived without incident, and no additional abnormal reactions were observed. Overall, the experimental animals demonstrated good tolerance to the conditions.

The primary pathological characteristic of obesity-related asthma is the imbalance between Th17 and Treg immune responses. The findings of this study demonstrated a significant increase in the expression of the Th17 cell-specific transcription factor RORγt and the cytokine IL-17A in the lung tissue of the fa group mice (36). Conversely, there was a decrease in the expression of the Treg cell core marker Foxp3 and the anti-inflammatory factor IL-10. These results indicate an overactivation of Th17 cells and an inhibition of Treg cell function, aligning with the mechanism by which obesity promotes a Th17-dominated T3 inflammatory transformation in asthma (37). Following intervention with XBCQD, these indicators were modulated in the opposite direction, with the low-dose group exhibiting the most pronounced improvement. This suggests that XBCQD can directly target the Th17/Treg balance, thereby reducing airway inflammation and remodeling by inhibiting Th17 cell activation and enhancing Treg cell function. These findings are consistent with previous research on the active components of XBCQD. For instance, chrysophanol in Da Huang has been shown to downregulate Th2 cytokines and inhibit the NF-κB pathway (38, 39), while amygdalin can modulate the TGF-β1/Smads pathway (40, 41), collectively contributing to the restoration of immune balance.

Intestinal microbiota dysbiosis serves as a critical link between obesity and asthma. In this study, sequencing data of the intestinal microbiota revealed a decrease in *α* diversity within the fa group, an increased abundance of *Firmicutes*, and a decreased abundance of *Bacteroidota* and *Verrucomicrobiota*, resulting in a significantly elevated *F/B* ratio. These findings align with the classical characteristics of obesity-related intestinal microbiota imbalance (42) and the observed trends in microbiota changes among asthma patients (43, 44). Following intervention with XBCQD, the aforementioned microbiota imbalance was rectified, with a notable increase in the abundance of SCFA-producing bacteria, including *Blautia*, *Dubosiella*, and *Akkermansia*. Subsequent research has demonstrated that beneficial bacteria, including *Bacteroidota*, *Lactobacillales*, *Akkermansiaceae*, *Alistipes*, and *Akkermansia_muciniphila*, are significantly associated with the amelioration of NLRP3, IL-1β, and Caspase-1, as well as the Th17/Treg immune imbalance. These bacteria are inversely correlated with airway resistance and obesity linked to obese asthma. Furthermore, studies have indicated a correlation between asthma and a reduction in the abundance of *Firmicutes* and *Bacteroidetes*. Our study additionally identified an increased *Firmicutes*/*Bacteroidetes* ratio in the intestinal microbiota of asthmatic mice (45). Moreover, research has shown that a long-term high-fat diet results in a decreased abundance of *Akkermansiaceae*, while *Lachnospiraceae* and Bacteroidaceae exhibit an increasing trend (46). The supplementation of *Akkermansia_muciniphila* has been shown to effectively mitigate LPS-induced lung injury by significantly decreasing oxidative stress and inflammatory responses. This intervention resulted in reduced levels of MDA, enhanced SOD activity, decreased pro-inflammatory cytokine levels, and diminished infiltration of macrophages and neutrophils (47).

In this study, montelukast sodium demonstrated a measurable effect in mitigating clinical symptoms and reducing airway inflammation in obese asthmatic mice; however, its efficacy in restoring intestinal flora diversity and enhancing the abundance of beneficial bacteria was limited. The potential for long-term use to fail in fundamentally correcting intestinal flora imbalance, which may lead to recurrent illness or the induction of other related complications, requires further investigation. Conversely, XBCQD exhibited a notable synergistic regulatory effect on both pulmonary and intestinal systems, effectively improving lung inflammation and airway function while also regulating intestinal microecological balance. Consequently, future research should focus on the development of prebiotics centered on traditional Chinese medicine and incorporate intestinal flora into the diagnostic index system for diseases such as obesity and asthma. This approach could provide novel insights and support for precise clinical diagnosis and treatment, ultimately reducing the risk of recurrence.

Correspondingly, the levels of acetic acid, propionic acid, and butyric acid in the serum of the treated mice increased. These data indicate that XBCQD facilitates the production of beneficial metabolites, specifically SCFAs, which act as key signaling molecules within the gut-lung axis by modulating the structure of the intestinal microbiota. SCFAs not only influence pulmonary immune cells via systemic circulation but also augment the function of regulatory Tregs by promoting Foxp3 acetylation and inhibiting DC activation (25, 48). This provides a mechanistic basis for XBCQD in modulating the Th17/Treg balance. These SCFAs are principal products resulting from the fermentation of substrates such as dietary fiber by intestinal microbiota. In healthy individuals, the total SCFA concentration in the colon is approximately 100 mM, with an acetate to propionate to butyrate ratio of about 3:1:1. Notably, over 90% of these SCFAs can be absorbed by the intestine, exerting both local and systemic regulatory effects. A decrease in intestinal pH confers a competitive advantage to butyric acid-producing bacteria over propionate-producing bacteria, resulting in an increased proportion of butyric acid in fermentation products as pH decreases (49). There are notable distinctions in the functions of various SCFAs, with butyrate being most closely associated with asthma outcomes. Empirical evidence indicates that butyrate supplementation can deactivate the p38 MAPK/NF-κB signaling pathway by binding to GPR43. This interaction inhibits Tfh13-mediated IgE production and suppresses the secretion of IL-4 and IL-13, thereby facilitating the restoration of CD4 + CD25^+^ Foxp3^+^ Treg cells in the spleens of OVA-sensitized mice. These mechanisms collectively result in a significant reduction of asthma symptoms (50). In contrast, acetate and propionate do not exhibit comparable effects (51). Furthermore, children exhibiting elevated concentrations of butyrate or propionate at the age of one demonstrate a reduced incidence of asthma by the age of six (52). However, it is important to note the functional complexity of butyrate, which, at physiological concentrations, can induce regulatory phenotypes. Conversely, elevated doses of butyrate may promote pro-inflammatory cell subtypes, such as Th17 and Th1, through the upregulation of IFNγ levels (53). Propionic acid exhibits anti-inflammatory properties by inducing and activating Treg cells through the upregulation of the transcription factor Foxp3. In contrast, acetic acid demonstrates a dual role: it may facilitate the differentiation of human monocytes into M1 inflammatory macrophages via the enhancement of p38 phosphorylation. Additionally, acetic acid can attenuate the expression of inflammatory cytokines such as C5, CCL1, and IL-1α through FFAR2 signaling, while also inhibiting Akt and ERK2 signaling pathways. Notably, the PI3K/Akt pathway is crucial in mitigating the pro-inflammatory response of TLR-stimulated macrophages, whereas the MEK/ERK signaling pathway is integral to oxidative burst and the activation of pro-inflammatory responses (54). In conclusion, the biological functions of various SCFAs exhibit considerable heterogeneity, with certain mechanisms remaining subjects of debate. Furthermore, their precise roles and regulatory networks warrant further investigation.

This study elucidates the comprehensive regulatory pathway of the “flora metabolite-receptor-inflammasome” axis. Following intervention with XBCQD, there was not only an increase in the levels of SCFAs but also an upregulation of GPR43, a receptor specific to SCFAs, in the lung tissue of obese asthmatic mice. Concurrently, the expression of the NLRP3 inflammasome and downstream pro-inflammatory factors, such as IL-1β and IL-6, was reduced. These findings corroborate previous mechanistic studies (55–57), which suggest that SCFAs can inhibit the activation of the NLRP3 inflammasome and decrease the release of pro-inflammatory factors by binding to GPR43 on the surface of immune cells, thereby mitigating airway inflammation. Additionally, the anthraquinone components of Da Huang in XBCQD, such as emodin and rhein, have been shown to directly inhibit NLRP3 inflammasome activation (58–60), acting synergistically with the regulatory effects of the gut microbiota. Collectively, these data confirm that XBCQD can attenuate obesity-related chronic inflammation through the “intestinal flora-SCFAs-GPR43-NLRP3” pathway, spanning from upstream intestinal microecology to downstream inflammatory pathways, thereby providing a molecular basis for the restoration of the Th17/Treg balance.

### Limitations of the study

The findings of this study elucidate, for the first time, the fundamental mechanism by which XBCQD modulates obese asthma via the gut-lung axis. This research not only substantiates the scientific basis of the “lung-gut correlation” theory in traditional Chinese medicine but also identifies a novel therapeutic target for the refractory phenotype of obese asthma.

Nonetheless, it is important to acknowledge the various limitations inherent in this study. Nevertheless, this study is subject to several limitations. Firstly, the proposed regulatory pathway involving “SCFAs-GPR43-NLRP3-Th17/Treg” is primarily based on correlational evidence and lacks direct causal validation. Key experimental mechanisms have not been investigated, including the application of GPR43 selective antagonists (such as CATPB) to inhibit receptor function, which would clarify whether the effects of SCFAs are dependent on GPR43-mediated signaling. Furthermore, specific inhibitors of NLRP3, such as MCC950-previously shown to influence the Th17/Treg balance in asthmatic mice-have not been employed, nor has an NLRP3 knockout model been utilized to confirm the pivotal role of the NLRP3 inflammasome within this pathway. Additionally, there has been no comprehensive analysis of the relationship between SCFA levels (particularly butyric acid and propionic acid) and the markers for GPR43, NLRP3, and Th17/Treg. Previous studies have established a linear correlation between SCFAs levels and GPR43 expression, yet the absence of direct evidence undermines the regulatory relationship among the components of this pathway. Third, it is not clear which active ingredient in XBCQD dominates the regulation of intestinal flora, and the pharmacokinetic mechanism of the optimal efficacy of the low-dose group is not explained. Fourth, mice treated with OVA and not subjected to a controlled diet were grouped for analysis and discussion.

Future research should prioritize the following areas: First, the causal relationships within the pathway should be verified using GPR43 antagonist interventions, MCC950 blocking experiments, and NLRP3 knockout models. Second, it is essential to supplement the correlation analysis between SCFAs and pathway molecules, as well as conduct exogenous SCFAs supplementation experiments, to elucidate the central role of bacterial metabolites. Third, the key active components of XBCQD in modulating intestinal flora should be identified through a combination of drug component separation and target verification. Fourth, it is recommended that OVA-treated mice be provided with a control diet in future studies, and these groups should be included in the discussion. Finally, pharmacokinetic studies should be conducted to elucidate the dose-effect relationship, providing a more direct reference for optimizing clinical dosages.

## Conclusion

In our study, we developed a model of obese asthmatic mice by combining a high-fat diet with OVA induction to investigate the therapeutic effects of XBCQD. Utilizing 16S *r*RNA gene sequencing, we explored the pathological mechanisms of obese asthma within the intestinal microbiota of these mice and examined the internal regulatory effects of XBCQD. Our findings demonstrated a significant association between obese asthma and intestinal flora imbalance, with XBCQD effectively reversing this dysbiosis. Specifically, XBCQD increased the abundance of SCFA-producing bacteria, including genera such as *Dubosiella*, *Bacteroidales*, *Bacteroidaceae,* and *Muribaculaceae*, and *Akkermansia*, by modulating the ratio of phyla *Firmicutes* to *Bacteroidota*. Additionally, it reduced the prevalence of bacteria associated with inflammatory responses, such as *Desulfovibrionales*, thereby promoting the generation of beneficial metabolites. Furthermore, XBCQD improved the Th17/Treg balance in the lungs via the SCFAs/GPR43/NLRP3 signaling pathway, effectively mitigating the symptoms of obese asthma. Our study elucidates the potential mechanisms by which XBCQD ameliorates obese asthma and offers empirical evidence supporting the use of XBCQD as a prospective therapeutic intervention for this condition.

## Data Availability

The datasets presented in this study can be found in online repositories. The names of the repository/repositories and accession number(s) can be found in the article/supplementary material.

## Footnotes

1
https://www.tcmsp-e.com/

2
https://pubchem.ncbi.nlm.nih.gov/

3
http://www.swisstargetprediction.ch/

4
https://www.uniprot.org/

5
https://www.genecards.org/

6
https://omim.org/

7
https://www.disgenet.org/

8
https://go.drugbank.com/

9
https://bioinfogp.cnb.csic.es/tools/venny/

10
https://cn.string-db.org/

11
https://www.bioinformatics.com.cn/

## Glossary

AKT1: Protein kinase B1
ASV: Amplicon sequence variant
BALF: Bronchoalveolar lavage fluid
BCL2: B-cell lymphoma 2 gene
CASP1: Caspase-1
CCL11: C-C motif chemokine ligand 11
CCND1: Cyclin D1
CTNNB1: Catenin beta 1
CXCR4: C-X-C chemokine receptor 4
EGFR: Epidermal Growth Factor Receptor
eNOS: Endothelial nitric oxide synthase
ERBB2: Epidermal growth factor receptor 2
ESR1: Estrogen receptor 1
FcεRI: High-affinity immunoglobulin E receptor
Foxp3: Forkhead box protein P3
GAPDH: Glyceraldehyde 3-phosphate dehydrogenase
GO: Gene ontology
GPR43: G protein-coupled receptor 43
HDAC: Histone deacetylase
HFD: High-fat diet
HSP90AA1: Heat Shock Protein 90 Alpha Family Class A Member 1
IgM/IgE: Immunoglobulin M/E
IL-33: Interleukin-33
ILC2: Type 2 innate lymphoid cells
JUN: Jun proto-oncogene
KEGG: Kyoto encyclopedia of genes and genomes
MAPK: Mitogen-activated protein kinase
MAPK3: Mitogen-activated protein kinase 3
MMP9: Matrix metallopeptidase 9
NF-κB: Nuclear factor kappa-light-chain-enhancer of activated B cells
NOD1: Nucleotide-binding oligomerization domain-containing protein 1
OVA: Ovalbumin
p-AKT: Phosphorylated AKT
PLCγ: Phospholipase C gamma
PPARG: Peroxisome proliferator-activated receptor gamma
PTGS2: Prostaglandin-endoperoxide synthase 2
Rorγt: Retinoic acid-related orphan receptor gamma t
SCFAs: Short-chain fatty acids
SRC: Src family tyrosine kinase
STAT3/STAT1: Signal transducer and activator of transcription 3/1
TC: Total cholesterol
TG: Triglyceride
TGF-β: Transforming growth factor-beta
Th: T helper
TLR4: Toll-like receptor 4
TNF-*α*: Tumor necrosis factor-alpha
TSLP: Thymic stromal lymphopoietin
VCAM-1/ICAM-1: Vascular/intercellular cell adhesion molecule 1
VEGFA: Vascular endothelial growth factor A
Wnt/β-catenin: Wnt signaling pathway/β-catenin
